# Marine mammals harbor unique microbiotas shaped by and yet distinct from the sea

**DOI:** 10.1038/ncomms10516

**Published:** 2016-02-03

**Authors:** Elisabeth M. Bik, Elizabeth K. Costello, Alexandra D. Switzer, Benjamin J. Callahan, Susan P. Holmes, Randall S. Wells, Kevin P. Carlin, Eric D. Jensen, Stephanie Venn-Watson, David A. Relman

**Affiliations:** 1 Department of Microbiology and Immunology, Stanford University School of Medicine, Stanford, California 94305, USA; 2 Veterans Affairs Palo Alto Health Care System, Palo Alto, California 94304, USA; 3 Department of Statistics, Stanford University, Stanford, California 94305, USA; 4 Sarasota Dolphin Research Program, Chicago Zoological Society, c/o Mote Marine Laboratory, Sarasota, Florida 34236, USA; 5 Translational Medicine and Research Program, National Marine Mammal Foundation, San Diego, California 92106, USA; 6 Space and Naval Warfare Systems Center Pacific, San Diego, California 92152, USA; 7 Department of Medicine (Infectious Diseases and Geographic Medicine), Stanford University School of Medicine, Stanford, California 94305, USA

## Abstract

Marine mammals play crucial ecological roles in the oceans, but little is known about their microbiotas. Here we study the bacterial communities in 337 samples from 5 body sites in 48 healthy dolphins and 18 healthy sea lions, as well as those of adjacent seawater and other hosts. The bacterial taxonomic compositions are distinct from those of other mammals, dietary fish and seawater, are highly diverse and vary according to body site and host species. Dolphins harbour 30 bacterial phyla, with 25 of them in the mouth, several abundant but poorly characterized Tenericutes species in gastric fluid and a surprisingly paucity of Bacteroidetes in distal gut. About 70% of near-full length bacterial 16S ribosomal RNA sequences from dolphins are unique. Host habitat, diet and phylogeny all contribute to variation in marine mammal distal gut microbiota composition. Our findings help elucidate the factors structuring marine mammal microbiotas and may enhance monitoring of marine mammal health.

## Abstract (web summary)

Little is known about the microbiota of marine mammals, despite the crucial ecological roles played by these animals. Here, Bik *et al*. describe the bacterial communities associated with various body sites in dolphins and sea lions, as well as the microbiota of their dietary fish and adjacent seawater.

## Graphical abstract

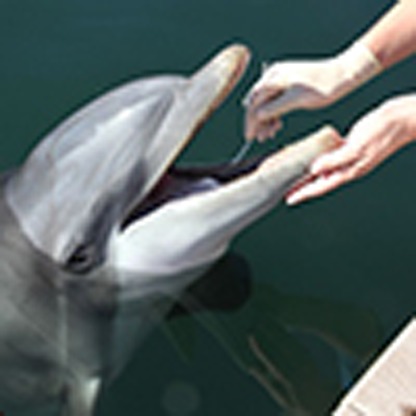

Marine mammals play essential roles in the marine ecosystem as apex predators, primary and secondary consumers, and indicators of ocean health^1^,^2^,^3^. Despite their importance, man has driven many marine mammal species to endangered status, and even extinction by hunting, overfishing and exploitation of their marine environment. Although conservation efforts have allowed some species to recover, many are still threatened, and there is an increasing need to better understand chronic, subclinical effects of ocean-borne threats on marine mammals and their populations' long-term survival and reproductive success^4^.

Mammal-associated microbial communities play critical roles in host nutrition, differentiation of host tissues, colonization resistance and the development of immune function, as well as other beneficial processes. Studies of faecal specimens from 60 terrestrial mammalian species have shown a striking degree of host specificity of microbiota, reflecting the influence of host phylogeny, gut anatomy, and diet^5^,^6^. Despite a growing understanding of the factors that shape the structure and function of the microbiotas of terrestrial animals, relatively little is known about those that influence the microbiotas of marine mammals.

Marine mammals provide an unusual opportunity to explore the relatedness of mammalian microbiotas as a function of evolutionary divergence. Extant marine mammal species descended from terrestrial ancestors that recolonized the sea and belong to one of three orders: Cetacea (whales, dolphins and porpoises), Sirenia (manatees and dugongs) or Carnivora (sea lions, seals, walruses, sea otters and polar bears). Cetaceans are derived from the group Cetartiodactyla^7^; the hippopotamus family (Hippopotamidae) represents the cetaceans' closest extant relatives^8^,^9^,^10^. Unlike hippopotamuses, cetaceans are carnivorous, and the diet of their most recent, now extinct, common ancestor is unknown^9^,^11^. The Carnivora suborder, Pinnipedia (sea lions, seals and walruses) is most closely related to the musteloids (raccoons, red pandas, skunks and weasels), with ursids (bears) as their sister group. Like their closest extant relatives, pinnipeds are carnivorous^12^. Members of Sirenia are most closely related to the Proboscidea (elephants), and both groups share a herbivorous diet. Although these marine mammal orders are phylogenetically distinct and their members exploit different food sources, all live and feed in an aquatic environment and therefore share many convergent adaptations^10^.

Several recent studies have explored marine mammal microbiotas, and provided important early assessments of microbial diversity in these critical host species^13^,^14^,^15^,^16^. Some culture-based studies have characterized gastrointestinal microbiotas of cetaceans^17^,^18^,^19^. Small scale, culture-independent studies have characterized the respiratory microbiota of dolphins^15^,^20^. Apprill *et al*.^21^ surveyed the skin microbiota of baleen whales. Distal gut microbiotas of seals^16^,^22^,^23^, dugong^24^, finless porpoise^25^ and baleen whales^26^ have also been surveyed. One study compared the microbiotas of terrestrial mammals with those of two species of seals and a dugong^14^; their results suggested that marine mammals harbour gut microbiotas that are more species rich than their terrestrial counterparts.

In this study, we analyse bacterial diversity in 337 specimens from 5 different body sites in 48 dolphins (order Cetacea) and 18 sea lions (order Carnivora), as well as adjacent seawater and dietary fish, and then compare our data with those from previously published studies of other animals. Our findings expand our understanding of bacterial diversity and microbial community structure in mammals and the influence of life in the sea, as well as enhance the means for monitoring and improving the health of marine mammals.

## Results

### A survey of marine mammal microbiota taxonomic composition

We examined bacterial phylogenetic diversity (PD) in the mouth, forestomach fluid (‘gastric fluid'), respiratory tract (blowhole swab and forceful exhalation referred to as chuff) and rectum of 38 common bottlenose dolphins (*Tursiops truncatus*; order Cetacea) and in the mouth, stomach and rectum of 18 California sea lions (*Zalophus californianus*; order Carnivora), from a group of healthy animals under the care of the US Navy Marine Mammal Program (MMP) in San Diego Bay, San Diego, CA (Supplementary Table 1), as well as the fish and squid that comprised their diet at this facility. In addition, we examined bacterial diversity in oral and rectal specimens from 10 healthy, wild bottlenose dolphins during capture-release health assessments in Sarasota Bay, Florida^3^. Seawater specimens were collected adjacent to each animal at the time of animal sampling, and processed and analysed together with the animal specimens. Using 2 different complementary amplification and sequencing approaches, a total of 20,030 near-full length (FL) Sanger-sequenced bacterial 16S ribosomal RNA (rRNA) gene amplicons and 915,150 pyrosequencing (PS) reads from the V3–V4–V5 region of the bacterial 16S rRNA were analysed, providing both high taxonomic resolution (FL sequences) as well as deeper community coverage (PS reads).

### High diversity within marine mammal microbiotas

**Figure 1:**
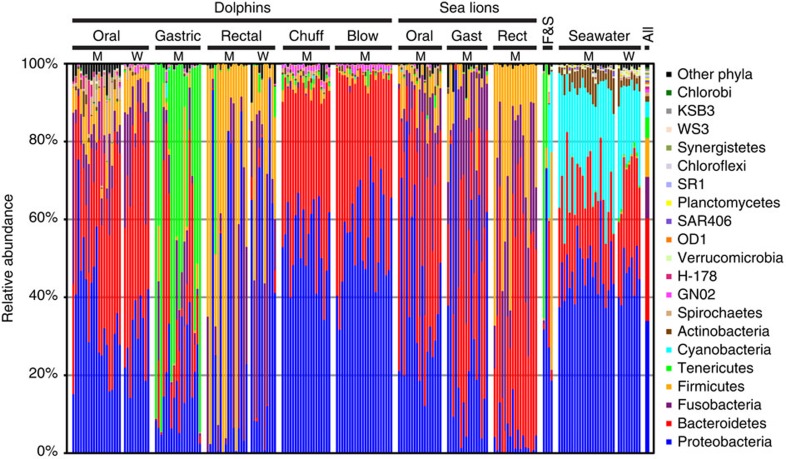
Bacterial phyla in specimens from 38 dolphins and 18 sea lions. Only one time point per animal and only specimens with ⩾372 pyrosequencing reads are shown (*n*=199, average number of reads per specimen was 3119.4). Four other single time point specimens and all 26 extraction controls yielded <50 reads. Specimens are shown in the same order as listed in Supplementary Table 1. The relative proportions of each phylum within each specimen and in the total data set are shown sorted on the average abundance in the combined data set (most abundant at the bottom). A total of 51 phyla were found. Only the 20 most abundant phyla are shown; the remaining 31 as well as all unclassifiable sequences were grouped together in ‘other phyla'; these are shown in Supplementary Fig. 1. Of the 20 phyla shown here, 19 were found in specimens obtained from the marine mammals; phylum SAR406 was only found in seawater samples. The horizontal lines at the top show the specimen source. ‘M' and ‘W' indicate specimens obtained from MMP and wild dolphins, respectively. No gastric or respiratory specimens were obtained from the wild dolphins, and in several cases different MMP dolphins were used for the respiratory sampling than for the oral/gastric/rectal sampling. Column ‘All' on the right displays the average relative phyla abundance in all specimens. Blow, blowhole; Gast, gastric; F&S, fish and squid.

Of all samples included in our study, seawater samples contained the highest phylum-level bacterial diversity: 48 phyla were detected with PS (Fig. 1, Supplementary Figs 1 and 2, Supplementary Data 1). Yet, representatives of 30 bacterial phyla were detected in the dolphin and sea lion microbiota specimens from the 5 body sites, based on PS reads (Fig. 1, Supplementary Fig. 1); 22 of these were also detected in the FL data set (Supplementary Figs 3 and 4a). All 30 phyla were found in the dolphin specimens alone, with oral, gastric fluid and chuff specimens containing the highest number of phyla (Supplementary Data 1). Most animal-associated and seawater specimens were dominated by Proteobacteria and Bacteroidetes, except for dolphin rectal specimens, which contained surprisingly few Bacteroidetes sequences (see below) (Supplementary Fig. 2). Dolphin gastric fluid specimens contained high relative proportions of Tenericutes, a phylum usually found at low abundance in mammal-associated microbiotas.

Representatives from 13 candidate phyla (phyla for which there are no laboratory-cultivated isolates) were detected in PS data from the marine mammal microbiotas (Fig. 1, Supplementary Fig. 1). Of these, GN02 was the most abundant, and was found primarily in dolphin oral (0.3% of PS reads), gastric (0.3%) and respiratory specimens (1.2%). About 1.9% of the dolphin oral reads were assigned to phylum H-178; all of these reads were detected in the MMP dolphins. This phylum was not found in the oral specimens of wild dolphins from Sarasota Bay, or in any of the sea lion specimens.

Seawater samples displayed the highest Operational Taxonomic Unit (OTU; species level) richness. Of the marine mammal-associated specimens, dolphin oral and respiratory specimens showed the highest number of observed OTUs, and dolphin rectal specimens the lowest (Supplementary Fig. 5).

**Figure 2:**
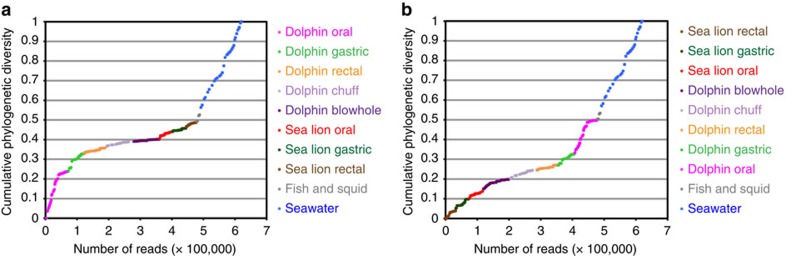
Gain in phylogenetic diversity according to specimen type. Phylogenetic diversity (PD) gain refers to the cumulative branch length in a phylogenetic tree that is exclusive to a particular specimen, as samples are added in a user-defined order. Calculations were done on the PS data set. Two different orders are shown. In **a**, the dolphin oral specimens were added first, followed by dolphin gastric, rectal and respiratory samples, sea lion specimens, fish and squid, and seawater samples. Panel **b** shows the animal samples in reversed order. Only one time point per animal was included. Of the marine mammal specimens, the dolphin oral specimens show the most phylogenetic gain, even when the sea lion specimens are considered first.

Among the marine mammal-associated specimens, the largest gain of PD (that is, the branch length a sample adds to a tree containing other samples) was found with the dolphin oral specimens, even when sea lion and other dolphin specimens were considered first (Fig. 2). Gains of PD were much lower in dolphin rectal and respiratory specimens, and sea lion gastric fluid and rectal specimens. Seawater samples yielded the highest gain in PD, even after addition of all marine mammal-associated sequences.

The FL sequence data set allowed a high taxonomic resolution analysis of bacterial novelty found in these marine mammals. The dolphin-associated FL sequences had a surprisingly high degree of novelty; overall, 70% of the dolphin-associated FL-OTUs (36% of sequences) and 44% of sea lion FL-OTUs (31% of sequences) were not similar to previously published sequences at a 97% sequence identity cutoff, with the greatest amount of novelty found in the dolphin oral cavity (Supplementary Fig. 6). At 90% identity, 4.0% of marine mammal-associated OTUs could be considered novel (Supplementary Data 2). These percentages are noteworthy in comparison to early studies of the human microbiota^27^,^28^,^29^ (Supplementary Fig. 6).

### Microbiotas from marine mammals and seawater are distinct

One possible explanation for the high degree of bacterial novelty in the two marine mammal species is their sharing or acquisition of bacterial taxa from seawater. To address this possibility, we compared the bacterial communities of sea lions and dolphins with the bacterial composition of seawater adjacent to these animals at the time of their sampling.

**Figure 3:**
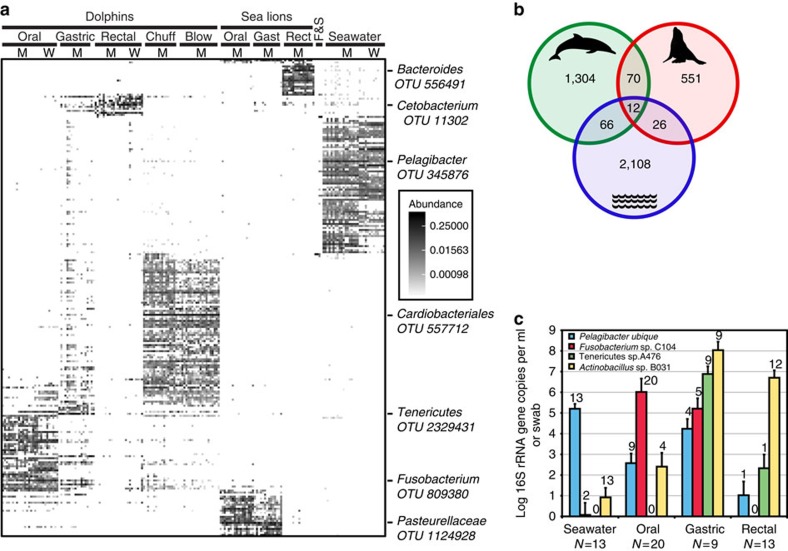
Habitat-specificity of bacterial groups found in this study. (**a**) Relative OTU abundance in the pyrosequencing data set. Specimens with ⩾372 reads (*n*=199; single time point per animal; same specimens and order as in Fig. 1) are shown in columns, and OTUs with ⩾20 reads (*n*=236) are shown in rows. The OTUs are clustered using an NMDS sorting based on Bray–Curtis distance, while the specimens (shown in columns) are sorted per specimen group. Relative abundance is shown in grey scale (white, absent; black, high abundance). (**b**) Venn diagram showing sharing of the 4,137 OTUs found in a rarefied pyrosequencing data set of 18 dolphins, 18 sea lions and 18 water specimens. Each data set was rarefied to 69,377 reads to match the smallest data set (from the water samples). Green, dolphins (1,452 OTUs total, includes oral, gastric, and rectal specimens from MMP and wild dolphins); red, sea lions (659 OTUs oral, gastric, rectal); blue, seawater (2,212 OTUs). The overlap between the circles is not to scale. (**c**) Quantitative PCR on four specimen types. Results are presented as average gene copy number per millilitre for seawater and gastric fluid, or per swab for oral and rectal specimens, for each of four different qPCR tests. Each individual qPCR was done in triplicate, error bars indicate s.d.. The numbers above the bars indicate the number of specimens positive for each qPCR test.

In both sequencing approaches, the bacterial community composition in seawater was profoundly different from that in the marine mammals (Fig. 3a, Supplementary Fig. 2). Despite the dominance of the phylum Proteobacteria at most body sites in dolphins and sea lions, as well as in sea water, Gammaproteobacteria were much more common in dolphins and sea lions, and Alphaproteobacteria much more common in seawater (Supplementary Fig. 4b). In particular, the 10 most abundant seawater-associated OTUs found by PS (PS-OTUs) together accounted for 39.1% of all seawater reads but for only 0.06% of the marine mammal-associated sequences (Supplementary Data 3). Most (278 out of 437) of the seawater-associated reads in marine mammal specimens were found in dolphin forestomachs, suggesting that some dolphins might have ingested seawater just before or at the time of sample collection. While the 10 most abundant dolphin rectum-associated PS-OTUs accounted for 81.1% of the dolphin rectum reads, their relative abundance in seawater collected next to these animals was only 0.05% (Supplementary Data 3).

About 95.3% of the bacterial PS-OTUs in seawater were not found in dolphins or sea lions, and 94.9% of the PS-OTUs found in the two marine mammals species were not found in seawater (Fig. 3b), based on a rarefied data set with equal numbers of PS reads and specimens representing dolphins, sea lions and water. The distinctness of the bacterial communities found in seawater and in dolphins was confirmed by quantitative PCR (qPCR) assays developed to detect bacterial groups highly abundant in each of three dolphin body sites and seawater (Fig. 3c). For example, *Pelagibacter ubique*, an abundant bacterium in most ocean surveys, was present at 10-fold lower concentrations in dolphin gastric fluid than in seawater collected next to the animals, and at >100-fold lower concentrations in dolphin oral specimens. Dolphin stomach and rectum-associated *Actinobacillus* sequences were detected at 1–10 million-fold lower abundances in seawater (Fig. 3c).

To investigate other potential sources of microbial diversity in marine mammals, we analysed the microbiotas of the fish and squid that are fed to the MMP dolphins and sea lions, but found these specimens contained distinct bacterial communities as well (Figs 1 and 3a, Supplementary Figs 2 and 4a). None of the fish- and squid-associated bacterial sequences were abundant in the marine mammals. The 10 most abundant PS-OTUs in fish and squid specimens together comprised 72.3% of reads from these specimens, but only 0.02% of all marine mammal-associated reads (Supplementary Data 3).

### Microbiotas differ among host species and body sites

**Figure 4:**
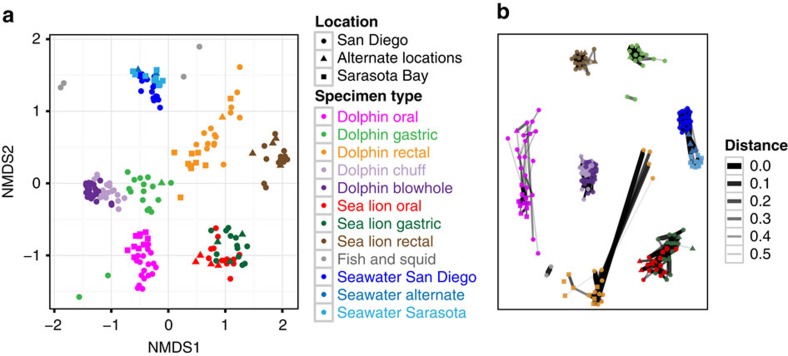
Relationships among bacterial communities from different host species and habitats. Only samples with ⩾372 reads were included (*n*=199; one specimen per body site per animal). (**a**) NMDS Bray–Curtis ordination of PS-analysed specimens from dolphins and sea lions included in this study. Colours indicate the different specimen types and sources. Shapes display the location of the animal during sampling (San Diego or an undisclosed alternate, distant location for the MMP animals and Sarasota Bay for the wild, free-living dolphins). (**b**) Distance Threshold Network analysis of marine mammal-associated bacterial communities. The network displays binary relationships between specimens and OTUs using a Bray–Curtis distance and a Fruchterman–Reingold layout. Each data point represents the bacterial community from an individual specimen. Two specimens are considered ‘connected' if the distance between them is less than a user-defined threshold, here, 0.6. The thickness of the connecting edge is related to the distance between two specimens. OTU abundances were proportionally transformed.

The composition of the indigenous microbiotas of dolphins and sea lions varied as a function of body site and host species (Fig. 4a, Supplementary Fig. 7). PS and FL read analysis portrayed very similar results in terms of the most abundant bacterial groups found among the sampled marine mammal body sites (Supplementary Fig. 2). Network analysis supported the role of host species and body site in explaining variance in community composition, and suggested close relationships between dolphin chuff and blowhole specimens, and between sea lion oral and gastric specimens (Fig. 4b). Interestingly, seawater bacterial communities from different oceans were more similar to each other than were communities from the same body site in different marine mammal species living in close proximity. No connections between the seawater samples and any of the marine mammal body sites were found.

No effect of age (2–51 years old) or sex on the composition of the dolphin microbiotas was apparent in ordinations, irrespective of sequencing approach (Supplementary Fig. 8).

The dolphin mouth showed high bacterial richness (Supplementary Fig. 5) and the highest PD (Fig. 2) and novelty (Supplementary Fig. 6). We detected 25 bacterial phyla in these specimens, with a predominance of Bacteroidetes and Proteobacteria, but a remarkably high number of additional phyla, including a surprising number of candidate phyla (*n*=11, 2.7% of PS reads) (Fig. 1, Supplementary Figs 1 and 2, Supplementary Data 1). In the FL data set with higher taxonomic resolution, OTU_C214 (Family Moraxellaceae, Class Gammaproteobacteria) was the most abundant (7.1%) and prevalent (found in 21 of 22 dolphins). Another abundant FL-OTU (3.6%) was C104 (95.0% identical to *Fusobacterium nucleatum*). This FL-OTU was found in 11 of 22 dolphin oral specimens and in all of 20 dolphin oral samples by qPCR (Fig. 3c). In addition, our study confirms the presence of a specific group of oral *Campylobacter*-like sequences in cetaceans^30^.

Twenty-two bacterial phyla were found in the dolphin forestomach, of which Tenericutes, Bacteroides and Proteobacteria were the most dominant (Supplementary Fig. 2). The most abundant FL-OTU, OTU_A476 in phylum Tenericutes (36.4% of forestomach-derived FL sequences) or PS-OTU 2329431 (40.2% of forestomach PS reads) was found in almost all (17 of 18) dolphin gastric fluid specimens. It is identical to a sequence found in the stool of the Yangtze finless porpoise^25^. The closest relatives of this sequence type (89% sequence identity) were found in the genus *Ureaplasma.* qPCR analysis revealed this sequence type in the forestomach of all dolphins in high abundance (10^7^ copies per ml gastric fluid), but not in dolphin oral specimens, sea lion-derived specimens or seawater (Fig. 3c). This sequence comprised 27.8% of dolphin forestomach PS reads (in 56 out of 69 gastric samples), 0.51% of dolphin rectal reads, <0.01% of dolphin oral, sea lion oral and gastric reads, and was not found among a total of 383,139 dolphin respiratory, sea lion rectal, fish, squid and seawater reads (Supplementary Data 3).

The microbial communities found in dolphin rectal specimens contained half as many bacterial phyla and four times fewer bacterial species as the dolphin oral specimens (Fig. 1, Supplementary Figs 2 and 5). Among the 12 bacterial phyla detected in the rectal specimens, Firmicutes, Proteobacteria and Fusobacteria were most abundant based on both sequencing approaches. The most abundant FL-OTU (29.3%) in dolphin rectal swabs, OTU_B031, was closely related to *Actinobacillus* sequences previously detected in cetaceans^25^,^31^. qPCR assays using specific primers detected this sequence in all gastric fluid specimens from both sea lions and dolphins (Fig. 3c) at high abundances, and in 4 of 20 oral specimens at approximately 10,000-fold lower abundances. A FL-OTU corresponding to *Cetobacterium ceti*^32^, OTU_K184, was the second most abundant (12.6%) among FL sequences and the most abundant (30.7%) among PS reads from dolphin rectal swabs (Supplementary Data 3). Of note, distinct *Arthromitus* sequences were found in 49 and 10% of dolphin and sea lion rectal specimens (via PS), respectively. Bacteria assigned to this genus in the Firmicutes phylum are also called segmented filamentous bacteria (SFB). They have been commonly detected in the gut communities of both invertebrates and vertebrates, with different *Arthromitus* clades detected in different hosts^33^. An analysis of the FL *Arthromitus* sequences found in our study confirmed the host specificity of these indigenous gut bacteria (Supplementary Fig. 9).

The bacterial communities of the two respiratory dolphin specimen types, chuff and blowhole swab, were very similar to each other, and dominated by Proteobacteria and Bacteroidetes. However, the chuff-derived PS reads were affiliated with a greater number of phyla (21) than a similar number of blowhole reads (11), as well as with more OTUs (Supplementary Figs 2 and 5). The dominant bacterial taxa in the bottlenose dolphin blowhole and chuff from our study were identical to those reported previously^15^,^20^. The most abundant PS-OTU in both the chuff (21.3%) and the blowhole (28.0%) was a member of the Cardiobacteriales, phylum Proteobacteria (Supplementary Data 3).

Based on data from both sequencing approaches, oral and gastric reads from sea lions were primarily assigned to the phyla Proteobacteria and Bacteroidetes (Supplementary Fig. 2). The most abundant taxon in both oral and gastric microbiotas of sea lions was a Pasteurellaceae related to *Actinobacillus porcitonsi*. *Neisseria canis* and several OTUs in Flavobacteria and Fusobacteria were also found in most of these specimens. The rectal communities of sea lions were dominated by Bacteroidetes, Firmicutes and Fusobacteria.

### Microbiota from free-ranging dolphins

**Figure 5:**
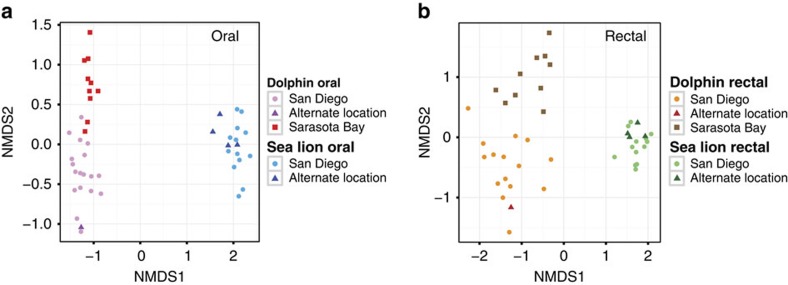
Oral and rectal bacterial communities associated with bottlenose dolphins and sea lions. Oral (**a**) and rectal (**b**) bacterial communities associated with bottlenose dolphins and sea lions. NMDS Bray–Curtis ordination of PS reads in which colour shows specimen type and data point shape depicts location (San Diego or alternate location for the MMP animals, Sarasota Bay for the wild animals). Only one time point per animal is shown. A bootstrap test in which the wild/MMP status of the dolphins was resampled showed that the average Bray–Curtis distance within oral specimens from MMP and within wild dolphins was significantly lower than the average distance between oral specimens from MMP and wild dolphins (*P*<0.001). However, average Bray–Curtis distances of dolphin rectal communities were not statistically different within or between location groups (*P*=0.11).

The composition of the oral microbiota from wild, free-ranging dolphins in Sarasota Bay, FL, was significantly different from that of MMP dolphins living in San Diego Bay, CA (*P*<0.001 bootstrap test, Fig. 5 and Supplementary Fig. 8). However, oral specimens were more similar between wild and MMP dolphins than either were to specimens from the same oral sites of MMP sea lions. In contrast, the differences between the rectal communities of wild and MMP dolphins did not reach statistical significance. Interestingly, *Fusobacterium* OTU_C104, an abundant OTU in the oral microbiota from dolphins, was nearly exclusive to MMP dolphins, whereas the abundance of *Fusobacterium* OTU_C718 was higher in the mouths of free-ranging animals (Supplementary Fig. 10). In addition, the oral microbiotas from MMP dolphins contained more Cardiobacterium, Fibrobacteres and Synergistetes sequences than those of wild dolphins, while the rectal microbiotas from wild dolphins contained more Campylobacteraceae sequences than those of MMP dolphins. However, because these two populations differ in geographic location, food sources and medical care, the clinical relevance of these differences remains unclear.

### Pathogens in dolphin and sea lion microbiotas

None of the bacterial pathogens typically associated with mortality in marine mammals^13^, for example, *Brucella* spp., *Erysipelothrix* spp. and *Streptococcus* group D, were detected in dolphins or sea lions, but *Staphylococcus aureus* was detected in multiple gastric fluid samples from dolphin Y and one from dolphin X. Several sequence types within the genus *Leptospira* were detected, but none close to *Leptospira interrogans*, a known pathogen in pinnipeds. One sea lion (SL08) carried an oral *Mycoplasma phocicerebrale*, which has been implicated in seal mortality and seal finger, a zoonosis among seal handlers^34^. Sequences closely related to certain respiratory pathogens in dolphins, such as *M. elephantis*, *Proteus mirabilis* and *Pseudomonas aeruginosa*, were found in dolphin chuff and blowholes in low numbers. In addition, sequences belonging to the genus *Helicobacter* were found in many stomach and rectum specimens from both dolphins and sea lions, most notably in the forestomach of dolphins (53 of 56 specimens; 2.7% of all dolphin gastric PS reads); none were found in oral or respiratory specimens. Several different clades within this genus were found, including *H. cetorum*, which has been previously reported in cetaceans and pinnipeds^30^,^35^, with different *Helicobacter* FL sequence types found within the same dolphin forestomach specimen (Supplementary Fig. 11). In addition to sequences closely related to *H. cetorum*, sea lion rectal specimens contained sequences from a clade most closely related to *Helicobacter canis*; sequences of this clade have been isolated from a variety of pinnipeds^36^. Because all animals included in our study were healthy, the clinical relevance of these *Helicobacter* spp. for cetacean and pinniped health remains unclear.

### Temporal stability of microbiotas at three body sites

**Figure 6:**
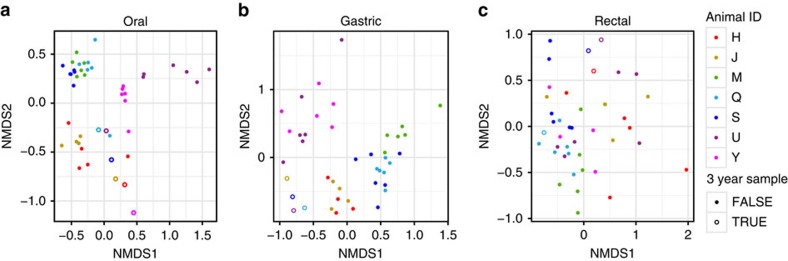
Relative stability of dolphin-associated microbial communities. (**a**) Oral communities, (**b**) gastric communities and (**c**) rectal communities. NMDS Bray–Curtis ordination of PS reads for each body site individually for the seven dolphins sampled monthly for 5 or 6 months. Six of these were sampled 3 years later as well (open circles). For each of the three body sites, intra-individual specimens were significantly more similar than interindividual specimens during the monthly sampling period (*P*<0.001, Mantel test). This heightened intra-individual similarity decayed or disappeared entirely at three years, although conclusions are limited by the small number of animals.

To gain insight into the temporal stability of the marine mammal microbiotas, seven MMP dolphins were sampled monthly over a period of 5–6 months, with an additional sampling after 3 years. As with the human microbiota^37^,^38^, dolphin oral, gastric and rectal microbiotas were relatively stable, with intra-individual variation significantly lower than interindividual variation at each body site during the monthly sampling (*P*<0.001, Mantel test). The oral microbiota was the most stable and distinct between animals on these timescales (Fig. 6). This stability appears to decay on longer timescales; the greater similarity of specimens sampled from the same dolphin was absent for oral and rectal communities and lower in gastric communities when specimens from the early monthly sampling period were compared with the specimen collected 3 years later.

### Integrated view of marine and terrestrial mammal microbiotas

To examine whether host adaptation to life in the sea influences the composition of host-associated microbiota, we compared oral, gastric and rectal communities of dolphins and sea lions to those of terrestrial and other marine mammals. First, we examined the relationships among oral microbiotas from dolphins (this study), sea lions (this study), humans^28^ and dogs^39^. Bray–Curtis distance analysis showed that oral communities clustered according to host species, with very little overlap in bacterial diversity between host species (Supplementary Fig. 12).

**Figure 7:**
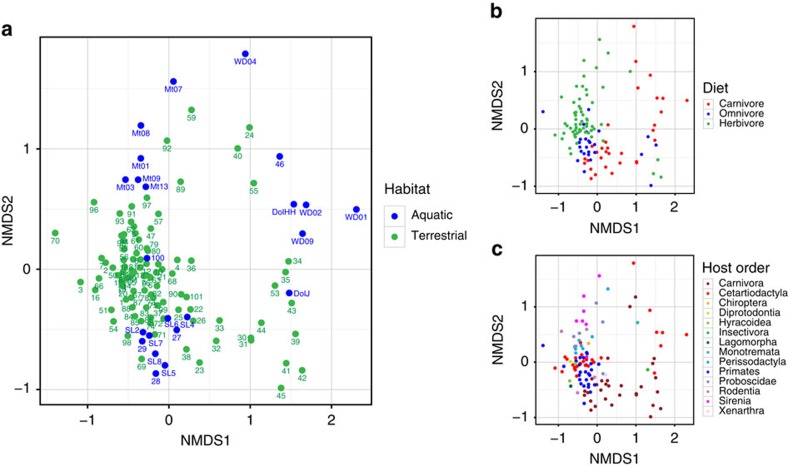
Comparison of distal gut communities obtained from different mammalian host species. (**a**) NMDS Bray–Curtis ordination of FL sequences from distal gut bacterial communities in 68 terrestrial and marine mammalian species, coloured according to host habitat. For these data sets, subsets of six individuals per host species (dolphins, sea lions and manatees) were chosen to provide a more balanced representation of marine mammal orders and diets. Specimens ‘100' (dugong^24^) and specimens starting with ‘Mt' (manatees, this study) were derived from herbivorous marine mammals (Order: Sirenia). Panels **b**,**c** show the same data as in **a**, coloured according to host diet (**b**) or mammalian phylogeny (Order) (**c**). See Supplementary Data 4 for more details on the individual data points and a key to the numbering shown in **a**. Supplementary Table 2 lists the most closely related communities to those from average dolphin and sea lion distal gut microbiotas.

To study the roles of host phylogeny, diet and habitat on the composition of host-associated microbiota, we compared the rectal microbiotas of sea lions, dolphins and manatees (this study), seals^16^, dugong^24^, river porpoises^25^ and polar bears^40^ with stool communities from 57 predominantly terrestrial mammalian species^5^,^29^,^39^,^41^,^42^,^43^,^44^ (Supplementary Data 4). In general, the distal gut microbiotas from marine mammals, in particular those of dolphins and manatees, were distinct from those of terrestrial hosts (adonis *P*=0.005) (Fig. 7a). Of note, the rectal microbiota of a dugong^24^, an herbivorous marine mammal from the order Sirenia, clustered with those of terrestrial Artiodactyl herbivores (Fig. 7b). Dolphin rectal communities were most similar to those of bears, especially polar bears, which spend time in or near the sea, and to those of the hooded seal, river porpoise, and interestingly, the omnivorous hedgehog (Supplementary Table 2). The clustering of the cetacean and bear gut microbiotas appeared to be driven by a lack of Bacteroidetes in both host groups (not shown). The rectal microbiotas of the carnivorous cetaceans were very distinct from those of herbivorous Artiodactyls (Fig. 7c), although both groups fall within the same order Cetartiodactyla^8^,^11^. The sea lion communities were most similar to those from the distal gut of three seal species and terrestrial members of the order Carnivora, including wolves and dogs (Supplementary Table 2).

**Figure 8:**
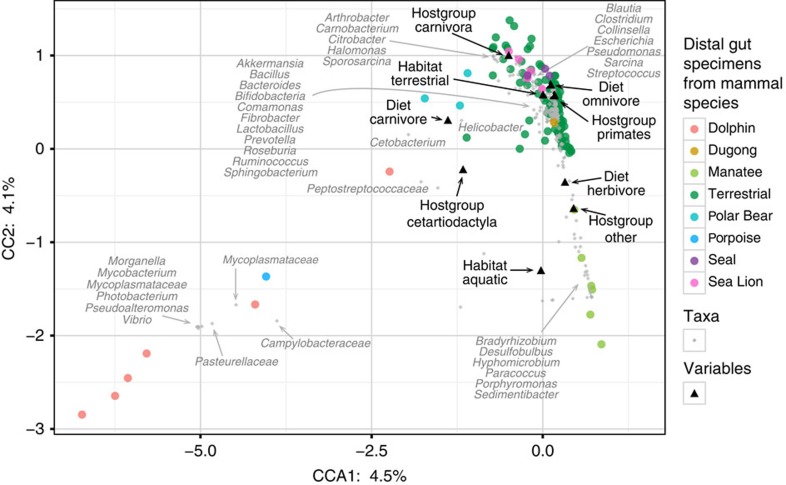
CCA of mammalian distal gut communities. Canonical Correspondence Analysis (CCA) plot showing the ordination of gut microbiotas from mammalian species (same as shown in Fig. 7, with subsets of six animals each for dolphins, sea lions and manatees) and bacterial taxa. Text in black displays the centroids of the three determinants that were tested, that is, habitat (aquatic and terrestrial), diet (carnivore, omnivore and herbivore) and host group (Carnivora, Cetartiodactyla, Primates and Other).

Canonical correspondence analysis (CCA) revealed that dolphin microbiotas were distinct from other mammals (Fig. 8). Despite the still limited number of marine mammal microbiotas available for analysis, these data are consistent with the conclusion that living in the sea is a strong determinant of distal gut microbiota taxonomic composition.

## Discussion

Marine mammals, positioned at the top of the marine food web, fulfil important ecological roles in the ocean and serve as sentinel species in many marine census studies^3^,^45^. While terrestrial mammalian hosts are the subject of an ever-increasing number of microbiome studies^5^,^6^,^46^ there have been relatively few studies of marine mammal microbiotas. Here we present a large-scale molecular analysis of the taxonomic composition of the bacterial communities associated with different body sites in marine mammals from two different clades. We surveyed the oral, rectal and gastric bacterial communities from dolphins and sea lions, as well as respiratory specimens from dolphins, using two different sequencing strategies. Although the archaeal, eukaryotic and viral components of the marine mammal microbiota were not determined here, we found a remarkable amount of previously unseen microbial diversity in these marine mammals, substantial differences between the microbial communities of dolphins and sea lions, and distinctness of the microbiotas of marine mammals from those of the surrounding aquatic habitat.

The microbiotas of dolphins in particular are characterized by high species richness, diversity and novelty. Representatives from 30 bacterial phyla were found in the dolphin specimens. By comparison, 22 phyla were found in a comparably sized data set of about 1 million reads from 6 body sites in humans^37^. A remarkably large number of bacterial phyla were found in the dolphin oral (25 phyla) and gastric (22 phyla) microbiotas alone. For comparison, sequences found in the human mouth^28^,^47^ and stomach^48^ were affiliated with only 15 and 9 phyla, respectively. Several candidate phyla were found in dolphin oral and respiratory microbiotas, such as GN02, H-178 and OD1. These phyla are most often found in environmental samples^49^, and their consistent presence in mammal-associated bacterial communities is noteworthy.

Marine mammal body sites defined specific bacterial communities with unexpectedly little overlap in OTUs. Our results are in accordance with large-scale studies of the human microbiome that highlight the primary importance of body site in explaining variation in bacterial diversity^37^,^50^,^51^. In our study, host species was also a strong determinant of community compositional variation, as illustrated by the distinctness of the dolphin and sea lion microbiotas, despite the MMP animals' common habitat (seawater), location (San Diego Bay), and diet (identical fish and squid species, dietary supplements and food sources). We found that the oral, gastric and rectal communities of individual dolphins were relatively stable over time, similar to what has been found for dolphin respiratory samples^20^. We did not find any patterns associated with age, sex or location.

The dolphin forestomach is dominated by a previously unclassified bacterial species, FL-OTU A476, assigned to the phylum Tenericutes. Since it is abundant to the forestomach of most MMP dolphins in our study, and found in the distal gut of wild dolphins and river porpoises^25^, we hypothesize that it might be specific for delphinoids, and possibly involved in the digestion of fish. Metagenomic analyses might clarify the metabolic properties of this bacterium. In addition to the A476 clade, most dolphin forestomachs contain one or multiple *Helicobacter* sequence types. All dolphins in our study were healthy without clinical signs of gastrointestinal disease; thus, the clinical significance of this finding remains unclear.

The microbial communities found in the dolphin distal gut differed from those found in other mammals in two interesting ways. First, unlike in humans where faecal communities are more diverse than oral communities^37^, the rectal communities of dolphins contained four times fewer bacterial species than their oral communities and added little PD to the overall dolphin microbiota. Second, dolphin rectal communities contained strikingly and yet unexplained low numbers of Bacteroidetes (<1%). Bacteroidetes are a major constituent of the distal gut microbiotas of most other mammals, including the dugong^24^, a herbivorous sirenian and carnivorous pinnipeds, for example, sea lions (this study and refs 16, 22, 23). The abundance at this body site of *Actinobacillus* and *Cetobacterium* spp., both of which have been described previously^25^,^31^,^32^, suggests that these species are common members of cetacean gut microbiotas.

Although all respiratory specimens from dolphins contained rich microbial communities, chuff contained higher phylum and species-level richness and diversity than the blowhole, suggesting that the deeper regions of the respiratory tract harbour more bacterial taxa than the upper regions, and probably provide a better representation of the lower respiratory tract microbiome than the blowhole. This finding has implications for clinical management since chuff is also a less invasive sample type.

Sea lion microbial communities were less diverse than those of dolphins. Their oral and gastric bacterial communities were similar in both phylum as well as species composition, and clustered more closely than dolphin oral and gastric specimens. This finding is somewhat similar to the situation in humans, where gastric and oral communities share many bacterial species^27^. Similar to the distal gut communities of most mammals except dolphins, sea lion rectal communities contained high numbers of Bacteroidetes and Firmicutes, and low numbers of Proteobacteria. However, their rectal specimens also harboured relatively high numbers of Fusobacteria, similar to what has been observed in seals^16^,^22^,^23^.

Rectal specimens from both dolphins and sea lions contained SFB (genus *Candidatus Arthromitus*). SFB are commensal bacteria anchored to the ileal mucosa and involved in the maturation of gut immune functions^52^. Specific *Arthromitus* clades have been detected in different hosts^33^, but they had not been detected in marine mammals prior to this study. Our study supports the host specificity of these indigenous intestinal bacteria.

Our study found a sharp boundary between microbial communities in marine mammals, their surrounding water and their fish and squid diet. This finding is all the more dramatic since the sea water samples examined in this study were collected immediately adjacent to each MMP and wild dolphin at the same time as animal specimen collection, and since sampling sites such as mouth and rectum of these animals are in constant direct contact with seawater. Similar distinctions have previously been found between earthworm-associated bacterial communities and those of their soil environment^53^, between the host-specific bacteria of marine sponges and those of their surrounding seawater^54^, and more generally, between those of terrestrial vertebrate gut microbiotas and those found in terrestrial environmental communities^55^. It is also a sharper boundary than typically found in landscape ecology, where patch boundary transitions are often more gradual^56^. Strong selective pressure exerted by the specific structures, conditions and immune surveillance mechanisms at mammalian mucosal surfaces may explain our findings. Yet, the marine mammal–seawater boundary is not absolute. Very low numbers of dolphin-associated OTUs were found in seawater, leaving open the possibility that marine mammals might be colonized in part through contact with seawater.

A more likely source of microbial seeding is contact between mother and calf. Our study included specimens from three mother–calf pairs but all three calves were ⩾2 years, and their communities did not cluster more closely to the adults than those of other dolphins. Sampling of mother and calf beginning at the time of birth, including vaginal samples of the mother, will be valuable for elucidating the role of vertical microbiota inheritance in marine mammals, and early life assembly of their microbiotas. Since dolphins are extremely social animals, it is also possible that microbial seeding occurs through physical contact between animals (for example, play, mating, and dominance displays such as raking)^2^.

While previous studies found that diet and host phylogeny are both important determinants of the composition of the mammal-associated microbiota^5^,^6^,^46^, our results suggest that habitat (aquatic versus terrestrial) is also an important determinant. The relatively distant ancestral relationships among extant marine mammals point to the importance of other shared adaptations to life in the sea that may have shaped the structure, and presumably the function of the marine mammal microbiotas. Further studies of bacterial, archaeal, eukaryotic and viral diversity in a broader and larger selection of marine and terrestrial mammal species with differing functional capacity, lifestyle and environment will be useful for elucidating the underlying relationships between indigenous microbial communities and their marine mammal hosts.

Understanding the composition of marine mammal microbiotas in healthy animals and how they are affected by environmental contaminants and changing prey profiles may provide valuable insight into potential long-term impacts of these increasing threats on marine mammal health. We expect that studies of this sort will help to define, predict and restore health in marine mammals^13^, and may provide a means for anticipating the impact of environmental change on ocean ecosystems by allowing for the surveillance of microbiome health in these sentinel species.

## Methods

### Animals included in this study

Forty eight common bottlenose dolphins (*T. truncatus*) (age range 2–51 years; 23 males) and 18 California sea lions (*Z. californianus*) (age range 1–27 years; all male) were studied (Supplementary Table 1). Thirty eight of the dolphins and all sea lions were managed by the US Navy MMP in San Diego, CA, USA. MMP dolphins and sea lions were housed in open-water, netted enclosures in San Diego Bay. At the time of sampling, one dolphin and four sea lions were housed at alternate locations in distant, distinct bodies of water. Both marine mammal species were fed mixtures of quality-controlled, frozen-thawed fish, including capelin (*Mallotus villosus*), herring (*Clupea pallasii*) and mackerel (*Scomber japonicas*), squid (*Loligo opalescens*) and additional vitamin supplements (Vita-Zu Mammal Tablet # 5M26, Mazuri, Richmond, Indiana, USA). All MMP-managed animals that participated in this study were assessed to be healthy by a veterinarian (for example, no gastrointestinal diseases or overt clinical signs), and had not received antibiotics or other medications within 30 days prior to sampling. The MMP is accredited by the Association for Assessment and Accreditation of Laboratory Animal Care (AAALAC) International and adheres to the national standards of the United States Public Health Service Policy on the Humane Care and Use of Laboratory Animals and the Animal Welfare Act. As required by the US Department of Defense, the MMP's animal care and use program is routinely reviewed by an Institutional Animal Care and Use Committee (IACUC) and the Navy Bureau of Medicine and Surgery (BUMED). In addition, 10 free-ranging, long-term resident bottlenose dolphins living in and near Sarasota Bay, Florida (South of Tampa Bay, in the Gulf of Mexico) were captured, examined, sampled and safely released by the Chicago Zoological Society's Sarasota Dolphin Research Program, conducted under the National Marine Fisheries Scientific Research Permit No. 552–1785 (ref. 3).

### Specimen collection

Collection of biological specimens is common practice in marine mammal veterinary care, and follows the guidelines set forth in the Chemical Rubber Company (CRC) Handbook of Marine Mammal Medicine^2^. Oral specimens were obtained from dolphins and sea lions by swabbing the gingival sulcus of the lower jaw with a sterile Fisherbrand Polyester-Tipped Applicator (Fisher Scientific, Pittsburgh, Pennsylvania, USA). Gastric fluid was collected by passing a small half inch outside diameter PVC stomach tube (JorVet, Jorgensen Laboratories, Loveland, Colorado, USA) into the forestomachs of MMP dolphins and stomachs of MMP sea lions, applying mild suction, then slowly removing the tube and draining the contents into a sterile 120 ml specimen container (Parter Medical Products, Carson, California, USA). The sample was then transferred into a sterile 15 or 50 ml polypropylene centrifuge tube (Corning, Corning Incorporated, Corning, New York, USA) prior to freezing. Rectal mucosa swab specimens were collected via sterile swab (same brand as above) passed through the anal orifice. Seawater was collected alongside sampled animals (MMP and free-ranging) at the time of animal sampling in a sterile 120 ml specimen container. Water samples were also transferred into sterile 50 ml polypropylene centrifuge tubes prior to freezing. Two types of respiratory specimens were collected from MMP dolphins: a blowhole mucosa swab (same brand as above) and a ‘chuff' specimen, for which the animal was signalled to forcefully exhale onto an 87 mm filter (Protran BA85, GE Healthcare Whatman, Dassel, Germany) placed above the blowhole. Specimens of fish (capelin, herring and mackerel) and squid, all of human-consumption quality, used to feed MMP dolphins and sea lions, were also collected. Fish and squid remained frozen at −20 °C until they were shipped on dry ice for processing. All other specimens were immediately placed under refrigeration, then processed and stored at −80 °C, and later shipped on dry ice for subsequent processing.

### DNA extraction

Seawater samples were prepared for DNA extraction by filtering the sample through a 150 ml 0.2 μm Nalgene analytical filter unit (cellulose nitrate filter, 47 mm diameter; Thermo Scientific, Waltham, Massachusetts, USA). After filtration, the filter was cut into pieces in a sterile Petri dish using a sterile scalpel, and placed into a sterile 2-ml screwcap vial (Sarstedt, Nümbrecht, Germany). Gastric fluid (1.5 to 2 ml) was centrifuged for 2 min at 15,000*g*, and the pellet was used for DNA extraction. Fish and squid specimens (one whole animal for herring, mackerel and squid, and about 100–200 gram of capelin pieces) were homogenized in 50 ml PBS in a Stomacher paddle blender (Seward Limited, West Sussex, UK) using two rounds of 60 s each on medium speed. Oral and rectal swabs were processed directly for DNA extraction using the QIAamp DNA mini kit (Qiagen, Valencia, CA; tissue protocol), according to instructions by the manufacturer, and then eluted in 200 μl AE buffer (supplied in the kit). During every extraction round, empty tubes (about 1 per 10 specimens) were processed in parallel to serve as negative extraction controls.

### Fish identification

The species identity of the fish used to feed the MMP dolphins and sea lions was confirmed by amplification and sequencing of the mitochondrial cytochrome B gene using primers CytB-F, 5′- AAAAACCACCGTTGTTATTCAACTA -3′ (ref. 57), and CytB-R, 5′- CGICCTCAGAAKGAYATTTGICCTCA -3′ (modified from ref. 57). One microlitre of the extracted fish DNA was added to an amplification reaction with 20 μl 2.5 × HotMasterMix (5 Prime Inc., Gaithersburg, MD, USA) and 0.4 μM of each primer, with a final volume of 50 μl. The PCR programme consisted of an initial denaturation at 94 °C for 2 min, 35 cycles of 95 °C for 30 s, 55 °C for 30 s, 65 °C for 30 s and a final incubation for 65 °C for 8 min. PCR products were purified using the QIAquick PCR purification kit (Qiagen), and Sanger-sequenced (Sequetech, Mountain View, CA). Comparison of the sequences using BLAST confirmed the fish species used for feeding the marine mammals to be *M. villosus* (capelin), *C. pallasii* (Pacific herring) and *S. japonicus* (chub mackerel), with 100% sequence identity to Genbank sequence entries DQ457494, KC588467 and KM2000011, respectively.

### Clone library construction and Sanger sequencing

To obtain near-FL (about 1,400 bp) 16S rRNA gene sequence information, the extracted DNAs from dolphin, sea lion, fish, squid and seawater specimens were amplified using broad-range bacterial-specific 16S rRNA gene primers, 8FMB (a 10:1 mixture of primers 8FM, 5′- AGAGTTTGATCMTGGCTCAG -3′ and *Bifidobacterium* spp.-specific primer 8FB, 5′- AGGGTTCGATTCTGGCTCAG -3′) and 1391R (5′- GACGGGCGGTGTGTRCA -3′)^58^. PCRs were performed as described previously^29^, using the following conditions: 5 min at 95 °C, 25 cycles of 30 s at 94 °C, 30 s at 55 °C and 90 s at 72 °C, followed by 8 min at 72 °C. No amplification product was observed in extraction controls and negative PCR controls. Because of eukaryotic background amplification issues, fish, squid and some wild dolphin specimens were amplified using primer mixture 8FMB (see above) and 806R (5′- GGACTACCAGGGTATCTAAT -3′)^27^, using a 30 s extension time and yielding an 800-bp fragment. After pooling four replicate amplification reactions, PCR products were purified from agarose gels using the QIAamp Gel Purification kit (Qiagen). Clone libraries were constructed with the TOPO TA cloning kit (Invitrogen, Carlsbad, CA). *Escherichia coli* TOP10 (Invitrogen) transformants were lysed and cloned inserts were amplified using M13 primers as directed in the cloning kit. PCR products were purified using the MultiScreen PCR_μ96_ Filter Plate (EMD Millipore, Billerica, MA), and sequenced from both ends using the T3 and T7 primers and Sanger sequencing technology (J. Craig Venter Institute, Rockville, MD). Forward and reverse reads were assembled using the Sequencher program (Gene Codes, Ann Arbor, MI).

### Phylogenetic analysis based on FL sequences

Assembled FL 16S rRNA gene sequences were aligned with the online Greengenes NAST aligner^59^ (http://greengenes.lbl.gov), and 20,852 sequences were uploaded into the Greengenes-version of the ARB database^60^,^61^. The alignment was further perfected by manual optimization. A total of 462 chimeras (2.2%) and 360 poor-quality reads (1.7%) were manually identified and removed from the analysis, leaving 20,030 high-quality, non-chimeric sequences derived from 77 specimens in the final analysis. Of these, 2,681 sequences were obtained from 6 seawater samples, 992 from 7 fish and squid specimens, 13,184 from dolphins (22 oral, 5 gastric and 19 rectal specimens), and 3,173 from sea lions (6 oral, 6 gastric and 6 rectal specimens). FL data sets consisted of an average of 260 sequences (range: 92–673) per specimen. Sequences were assigned to bacterial phyla using the SILVA release 102, Ref-NR ARB database^62^ and the Ribosomal Database Project (RDP) Release 10 Classifier^63^. OTUs (FL OTUs) were defined using a 99% sequence similarity cutoff, using a 1,256- or 684-nucleotide mask on the PCR products generated by reverse primers 1391R or 806R, respectively, filtering out the hypervariable regions. The 99% cutoff in this setting roughly corresponds to species-level groupings. The FL sequence OTU table is available as Supplementary Data 5. Phylogenetic neighbour-joining trees were created using the Jukes Cantor correction and the 1256-nt filter. Additional alpha- and beta-diversity analyses were performed with the R package phyloseq^64^. The R code is available in Supplementary Software 1.

### Determination of sequence and taxa novelty

The percentage of novel OTUs found in the FL sequence data set, using different levels of sequence identity, was determined by comparing the sequences of OTU representatives to published sequences using BLAST. Novelty of the OTUs from dolphin and sea lion oral, gastric and rectal FL data sets (this study) was compared with the novelty of human oral sequences^28^, human gastric sequences^27^, and human rectal biopsy and stool sequences^29^ at their time of publication. These previously published data sets were chosen since they were all amplified and analysed by nearly identical methods. All FL sequence sets were rarefied to 1,013 sequences and 6 individuals per host species, except the human intestinal samples, for which 6 specimens from a total of 3 individuals were used, and the human gastric samples for which 15 individuals were chosen. For each FL OTU obtained in this study, a BLAST search was performed using the standard parameters and excluding the primer positions, and the percentage identity of that OTU to its top hit in the NCBI nt database (version March 2015) was used as the outcome. For the human sequences, the BLAST identity percentages obtained at the time of publication were used.

### Comparing oral microbiotas from different mammalian species

FL oral 16S rDNA sequences derived from 12 MMP and 10 wild dolphins (7,149 reads) and 6 MMP sea lions (1,050 reads) obtained in this study were compared with FL 16S rDNA sequences from dogs and humans. The canine oral microbiota data set^39^ consisted of 4,253 sequences from 51 dogs (294 OTUs); only sequences from broad-range bacterial amplification reactions were included in our analysis. The human oral microbiota data set^28^ included 10,285 sequences obtained individually from 10 humans with healthy mouths (241 OTUs). A neighbour-joining tree containing representatives of the 1,465 OTUs found in the combined data set was built using *Halobacterium salinarum* as the outgroup. The 89 microbial communities were compared with each other using the Bray–Curtis distance and NMDS ordination in the phyloseq R package^64^. The R code is available as Supplementary Software 2.

### Comparing gut microbiotas of terrestrial and marine mammals

FL rectal sequences from 9 MMP and 10 wild dolphins and 6 MMP sea lions (this study) were compared with published distal gut data sets derived from 66 terrestrial and marine mammal species from other studies. First, sequences derived from stool specimens of 57 different terrestrial mammals^5^ were downloaded from SILVA release 102 (http://www.arb-silva.de); only sequences with a Pintail value >75% were selected to obtain a data set of 19,335 sequences. In addition, we included sequences obtained from faeces of a Yangtze finless porpoise^25^, colon contents from grey seals, harbour seals and hooded seals^16^, pooled polar bear faeces^40^, the faeces of a giant panda^44^, colon of domestic dogs^65^, faeces of a dugong^24^, caecal contents of lean mice^41^ and faeces of wolves^43^. We also included stool specimen sequences from three healthy humans^29^ and stool specimen sequences from three healthy humans prior to antibiotic exposure^42^. Finally, to add more representatives from the aquatic herbivorous Order Sirenia, we added sequences from rectal samples of wild manatees (Florida) obtained by PS. The total data set, including sequences obtained in this study, consisted of 46,321 16S rRNA sequences derived from 68 different mammalian species (135 individuals) (Supplementary Data 4), and was analysed using the Quantitative Insights Into Microbial Ecology (QIIME) software package^66^. In short, OTUs in the combined mammalian gut data set were picked against a reference database using a similarity threshold of 97% and the UCLUST package^67^, and OTU representatives were aligned and assigned to taxonomic groups against the Greengenes core set alignment^61^. OTUs were grouped into genera, and an NMDS ordination on Bray–Curtis distance was constructed with phyloseq^64^ and used to explore beta-diversity. CCA was run on OTU tables using the vegan R package and plots were constructed displaying specimens, OTUs, and centroids representing the relative contribution of the environmental variables (habitat, diet and host taxonomy) considered as factors. To provide a more balanced representation of the marine mammal orders, subsets of six dolphins and six manatees were included in the CCA; all six sea lions were kept in this analysis. The R code is available as Supplementary Software 3.

### PCR amplification for PS analysis

Extracted DNA from specimens derived from 36 dolphins and 18 sea lions was used for PS. A 589-bp fragment of the bacterial 16S rRNA gene spanning the hypervariable V3–V4–V5 regions was amplified. The forward primer (5′- CGTATCGCCTCCCTCGCGCCATCAG-NNNNNNNNNNNN-GC-ACTCCTACGGGAGGCAGCA -3′) contained the 454 Life Sciences primer-A sequence, a unique 12-nt error-correcting Golay code (a different barcode for each specimen; depicted by the Ns in the sequence)^68^, a two-base linker sequence (‘GC') and the broad-range bacterial primer 338F. The reverse primer (5′- CTATGCGCCTTGCCAGCCCGCTCAG-AA-CCGTCAATTCCTTTGAGTTT -3′) contained the 454 Life Sciences primer-B sequence, a two-base linker (‘AA') and the broad-range bacterial primer 906R. One to 2.5 μl of extracted DNA were added to a 25 μl amplification reaction containing HotMasterMix and 0.4 μM of each primer. The PCR programme consisted of an initial denaturation at 94 °C for 2 min, 25 cycles of 95 °C for 30 s, 53 °C for 30 s, 65 °C for 30 s and a final incubation at 65 °C for 8 min. Three or four replicate PCR mixes were pooled, and the amplicons were analysed on agarose gels. In case of weak amplification reactions, an additional 4 replicate PCRs (30 cycles in cases of very low yield) were performed and pooled. PCR amplicons were purified using the QIAquick PCR purification kit or the UltraClean-htp PCR clean-up kit (MoBio, Carlsbad, CA). DNA concentrations were determined using the Quant-iT High Sensitivity DNA Assay kit (Invitrogen). Equimolar amounts of the barcoded PCR products were pooled, ethanol precipitated and gel-purified using the QIAquick Gel Extraction kit (Qiagen). The purified pool was sequenced using the 454 Life Sciences Genome Sequencer FLX Titanium platform (Roche, Branford, CT).

### Processing and analysis of PS reads

A total of 337 specimens and 26 extraction controls from this study were analysed in three PS runs. Reads were processed using the QIIME software package^66^. In short, reads from each sequencing run were quality-filtered (quality score 25, length >200 and <1,000 nt, no ambiguous bases, homopolymer length <6), assigned to their original source specimen using the unique barcodes and primer sequences were removed. Denoising was performed in specimen type-specific batches of roughly 10,000 reads each, using the denoise_wrapper.py command within QIIME. Chimeras were identified and removed using UCHIME^67^ using a customized reference database derived from the Greengenes 12–10 release clustered at 99% sequence identity threshold (available on request); 14.7% of reads were removed by this step. Sequences were clustered into OTUs using a similarity threshold of 97% against the same reference database as used for UCHIME and allowing for new clusters. OTU-representative sequences were aligned and masked using the Lane mask, and a phylogenetic tree was built using the FastTree software implemented in QIIME. Taxonomy was assigned to each OTU-representative sequence using the RDP classifier, a curated Greengenes reference database (see above), and a confidence score of at least 80%. In the data set used in this study, 8,117 OTUs were found among 915,150 reads (including 26 extraction controls, which combined yielded 180 reads). The PS data OTU table is available as Supplementary Data 6. Not counting the extraction controls, the average number of reads per specimen was 2,715 (range 0–14,184).

### Analysis of bacterial diversity from PS data

Bacterial diversity within specimens (alpha-diversity) or between specimens (beta-diversity; using Bray–Curtis distances) was calculated using QIIME^66^ and the R package phyloseq^64^, and a non-rarefied data set from samples with 372 reads or more. The R code for the PS data set is available as Supplementary Software 4.

### Calculation of gain in PD

The gain in PD is the branch length a sample adds to a tree containing other samples. It is the branch length exclusive to that sample^69^,^70^. The gain in PD depends on the other taxa already present in the tree, so the order in which samples are added plays an important role. PD gain was calculated in QIIME^66^ by using the ‘unifrac_g_full_tree' parameter executed in the beta_diversity.py script. Using the fraction of branch length in the full tree that is exclusive to a particular specimen, it calculates the PD on cumulative OTU tables, where each specimen is added to the calculation in a user-defined order. For the exploration of marine mammal-associated bacterial diversity, two different orders of sample addition were tested.

### Temporal stability of bacterial communities in dolphins

To test the temporal stability of bacterial communities within the same animal, seven dolphins were sampled monthly for 5 or 6 months at the gastric, oral and rectal anatomical sites. Six of these animals were sampled again 3 years later. Pairwise Bray–Curtis distances were calculated between all specimens from the same anatomical site, based on PS reads. A Mantel test was used to determine whether samples from the same dolphin were more similar than were samples from different dolphins during the monthly sampling period. To be specific, a Mantel test was performed on the matrix of Bray–Curtis distances and the distance matrix obtained by assigning 0 to pairs of samples from the same dolphin and 1 to pairs of samples from different dolphins. The late sample (after 3 years) was treated separately: The average distance between late samples and monthly samples was calculated for samples from the same and different dolphins, and compared with the same- and different-dolphin averages among monthly samples. Because of the small number of animals and the single late sample, we make no significance statements about this comparison. These calculations were performed in R, and are available in Supplementary Software 4.

### Shared OTUs in different specimen types

Shared OTUs between dolphin, sea lion and seawater were identified using a rarefied PS data set; data were selected from 18 (12 MMP and 6 wild) dolphins (120,527 reads), 18 sea lions (116,167 reads) and 18 seawater samples (13 samples taken alongside MMP dolphins or sea lions and 5 alongside wild dolphins; overall 69,377 reads). All three data sets were rarefied to 69,377 reads in QIIME^66^. The number of OTUs that were shared between or unique for specimen types was plotted in a Venn diagram. Fish specimens were not included in the analysis because not all fish used to feed the MMP animals yielded PS reads, and because diet samples were not available for wild dolphins.

### Quantitative PCR

To analyse the presence of specific bacterial groups that were abundant in FL sequence libraries derived from particular specimen types but were absent or rare in FL data sets from other habitats, four specific qPCRs were developed. In short, qPCRs were developed for the detection of OTU_O614, *P. ubique*, abundant in seawater; *Fusobacterium*-like OTU_C104, abundant in dolphin oral specimens; Tenericutes sp. OTU_A476, abundant in dolphin forestomachs; and OTU_B031, abundant in dolphin rectal specimens. Primers and probe sequences are provided in Supplementary Table 3. All qPCR assays were performed in 20 μl reactions containing 1 × TaqMan Universal PCR Master Mix (Applied Biosystems), 0.9 μM of each primer, 0.2 μM of the probe and 2 μl of extracted DNA. The thermal cycling programme consisted of 95 °C for 10 min, followed by 40 cycles of 95 °C for 30 s, 55 °C for 30 s, 60 °C for 45 s, 65 °C for 15 s, 72 °C for 30 s. All reactions were carried out in triplicate on a StepOnePlus Real Time PCR System (Applied Biosystems). Ten-fold serial dilutions of known quantities of plasmids containing the specific sequence target for each assay were used to generate standard curves. Sequence abundance was calculated using SDS software version 2.1 (Applied Biosystems).

## Additional information

**Accession codes:** All high-quality, FL 16S rRNA gene sequences obtained in this study have been deposited in the GenBank Nucleotide database with accession codes JQ191150 to JQ217071 and KC257481 to KC260956. The 16S rRNA gene pyrosequencing data have been deposited in the NCBI's BioProject database with accession codes PRJNA174530 (oral, gastric, rectal and water sequences) and PRJNA279427 (respiratory and additional water sequences).

**How to cite this article:** Bik, E. M. *et al*. Marine mammals harbour unique microbiotas shaped by and yet distinct from the sea. *Nat. Commun.* 7:10516 doi: 10.1038/ncomms10516 (2016).

## Supplementary Material

Supplementary Information Supplementary Figures 1-12, Supplementary Tables 1-3 and Supplementary

Supplementary Software 1 R code for analysis of FL sequences obtained from dolphins, sea lions, fish food, and seawater.

Supplementary Software 2 R code for comparison of FL oral sequences obtained in our study to those from humans and dogs.

Supplementary Software 3 R code for comparison of distal gut FL sequences obtained from dolphins, sea lions, to those from published datasets.

Supplementary Software 4 R code for analysis of PS sequences obtained from dolphins, sea lions, fish food, and seawater.

Supplementary Data 1 Fractional abundances of bacterial phyla detected in specimens analyzed in this study.

Supplementary Data 2 Novel OTUs found in marine mammal specimens, with less than 90% identity to published sequences, based on FL data.

Supplementary Data 3 Most abundant OTUs for each specimen type, based on the pyrosequencing data.

Supplementary Data 4 Data included in the comparison of distal gut communities from terrestrial and marine mammals performed in this study. These data are also presented in Figs. 7 and 8.

Supplementary Data 5 OTU table of near full length (FL) sequences.

Supplementary Data 6 OTU table of pyrosequencing (PS) reads.
