# Supplementary material for: Marine mammals harbor unique microbiotas shaped by and yet distinct from the sea
Source: Nat Commun. 2016 Feb 3;7:10516. doi: 10.1038/ncomms10516 (PMC4742810; doi:10.1038/ncomms10516)
Supplement: Supplementary Information — Supplementary Figures 1-12, Supplementary Tables 1-3 and Supplementary [file ncomms10516-s1.pdf]

Supplementary Figure 1

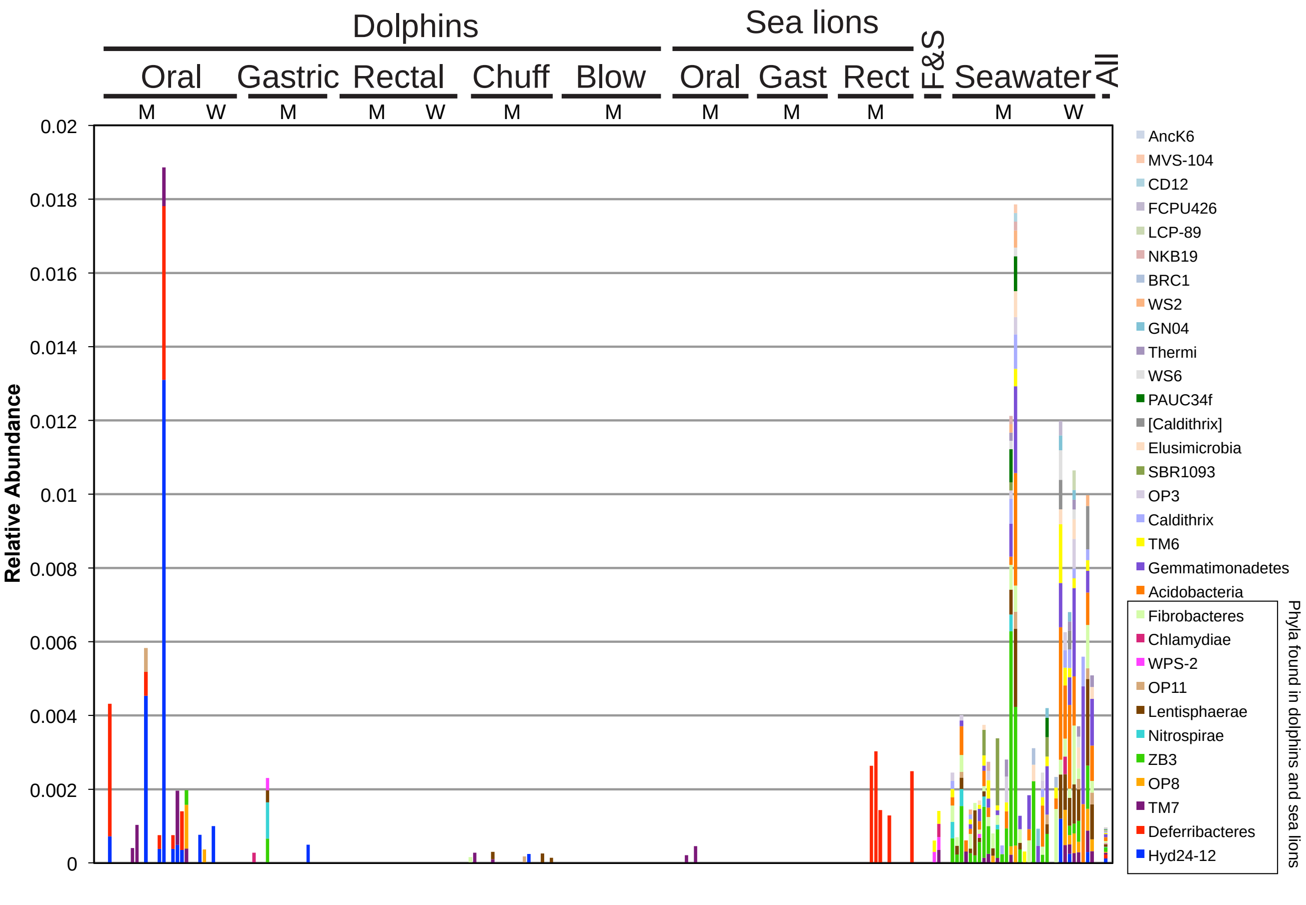

Supplementary Figure 1. Relative abundance of rare phyla based on pyrosequencing data. Relative abundances of the 31 low-abundance phyla that were grouped as “Other” in Fig. 1 is shown here. Phyla that were found in specimens derived from dolphins and sea lions are shown in a box; the other phyla were found in seawater samples only. F&S, fish and squid; Blow, blowhole; Gast, gastric fluid; Rect, rectal; All, all specimens combined; M, MMP animals; W, wild dolphins.

Supplementary Figure 2

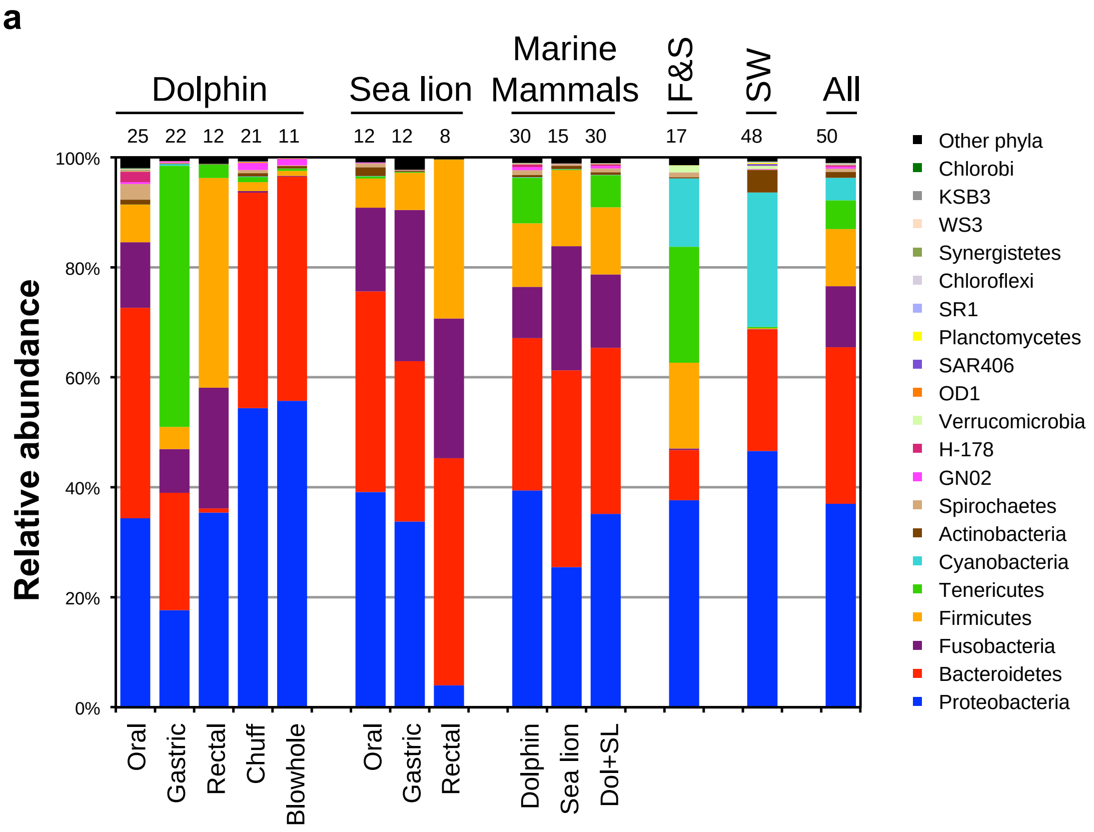

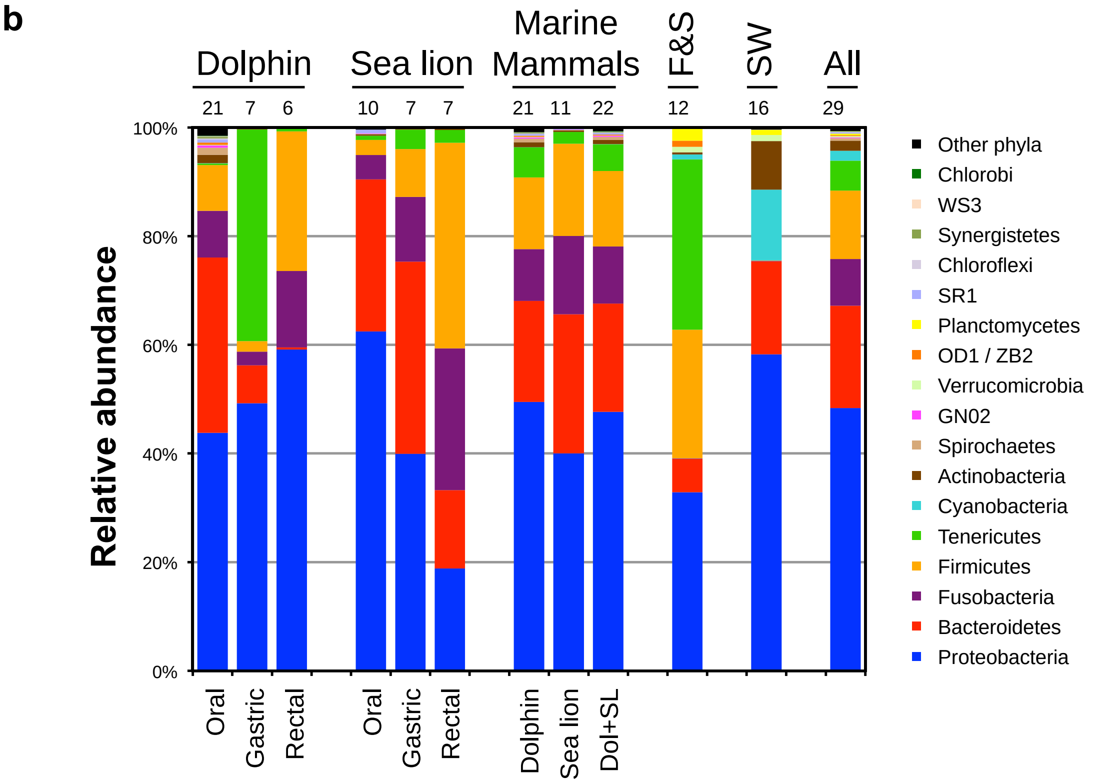

Supplementary Figure 2. Phylum distribution for different specimen types based on pyrosequencing (a) or near-full length (b) data. Single timepoint specimens were grouped according to specimen type. The relative proportions of sequences assigned to each phylum within each group are displayed. Only the most abundant phyla are shown; all other phyla are grouped together. The exact proportions of all phyla found within each specimen type are available in Supplemental Data 1. Numbers at the top of each column indicate the numbers of phyla found in that specimen type. Dol, dolphin; SL, sea lion; F&S, fish and squid; SW, seawater; ALL, all sequences combined.

Supplementary Figure 3

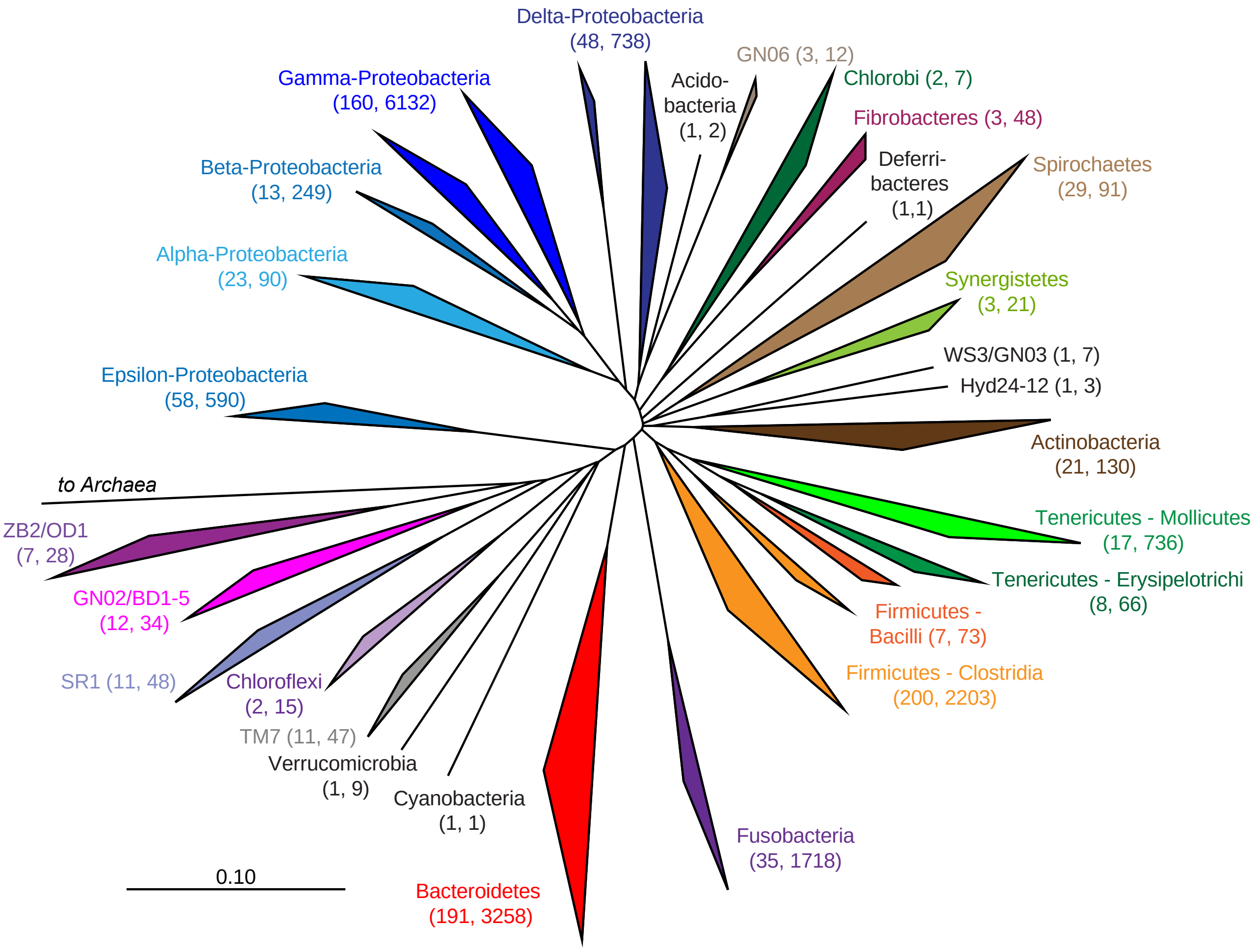

Supplementary Figure 3. A phylogeny of phylum-level groups in the marine mammal microbiota based on FL sequences. Neighbor-joining tree built in ARB[1] with the 870 OTUs (99% cutoff) found in 16,357 FL bacterial 16S rRNA sequences derived from 12 MMP dolphins, 10 wild dolphins, and 6 MMP sea lions. OTU representative sequences were grouped into phyla, or (in case of Firmicutes, Tenericutes, and Proteobacteria) into classes. A total of 22 phylum-level clades was found. The two numbers in parentheses represent numbers of OTUs and sequences, respectively, within that labeled branch of the tree. The scale bar represents 10% sequence distance.

Supplementary Figure 4

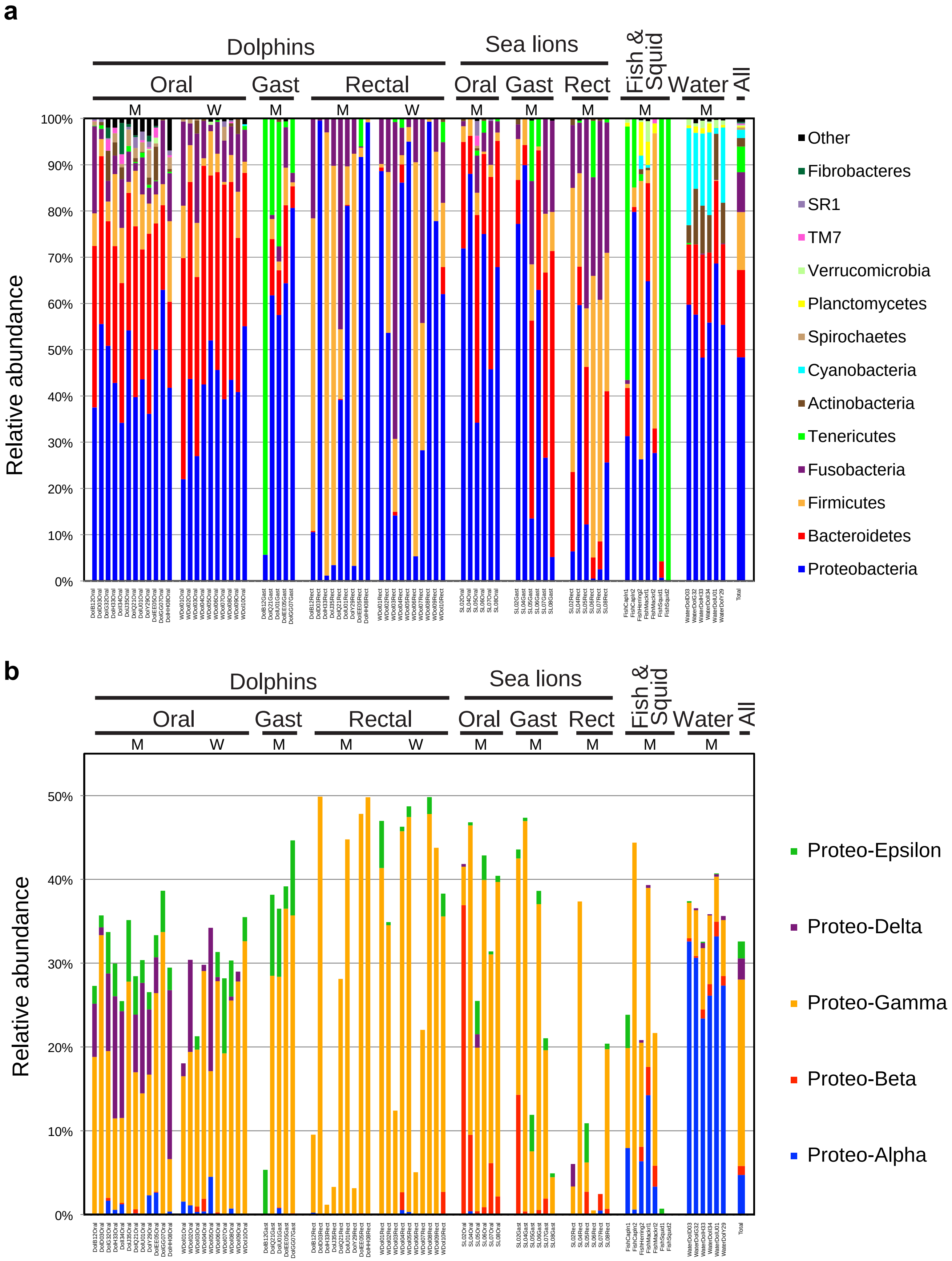

Supplementary Figure 4. Relative abundances of phyla (a) or Proteobacteria class (b) in the marine mammal microbiota, based on FL sequences. The relative proportion of sequences within each specimen and in the total FL dataset (20,030 sequences) are shown, color-coded according to taxonomic group. Specimen names indicating animal species, ID, and specimen type are shown at the bottom. The FL dataset did not contain respiratory samples. Only one timepoint per animal is shown. Gast, gastric; Rect, rectal; All, all specimens combined; M, MMP animals; W, wild dolphins. **a**. Relative abundance of phyla. Only the 13 most abundant phyla are shown; 19 other phyla are grouped together and shown as “Other phyla”; their abundances per specimen group can be found in Supplementary Data 1. **b.** Class distribution of the 9,687 sequences within the Proteobacteria phylum. The relative proportion of FL sequences within each specimen and in the total dataset are shown, color coded according to Class. Individual animal letters or numbers are shown at the bottom. One of the squid samples did not contain any Proteobacteria. Proteo, Proteobacteria.

Supplementary Figure 5

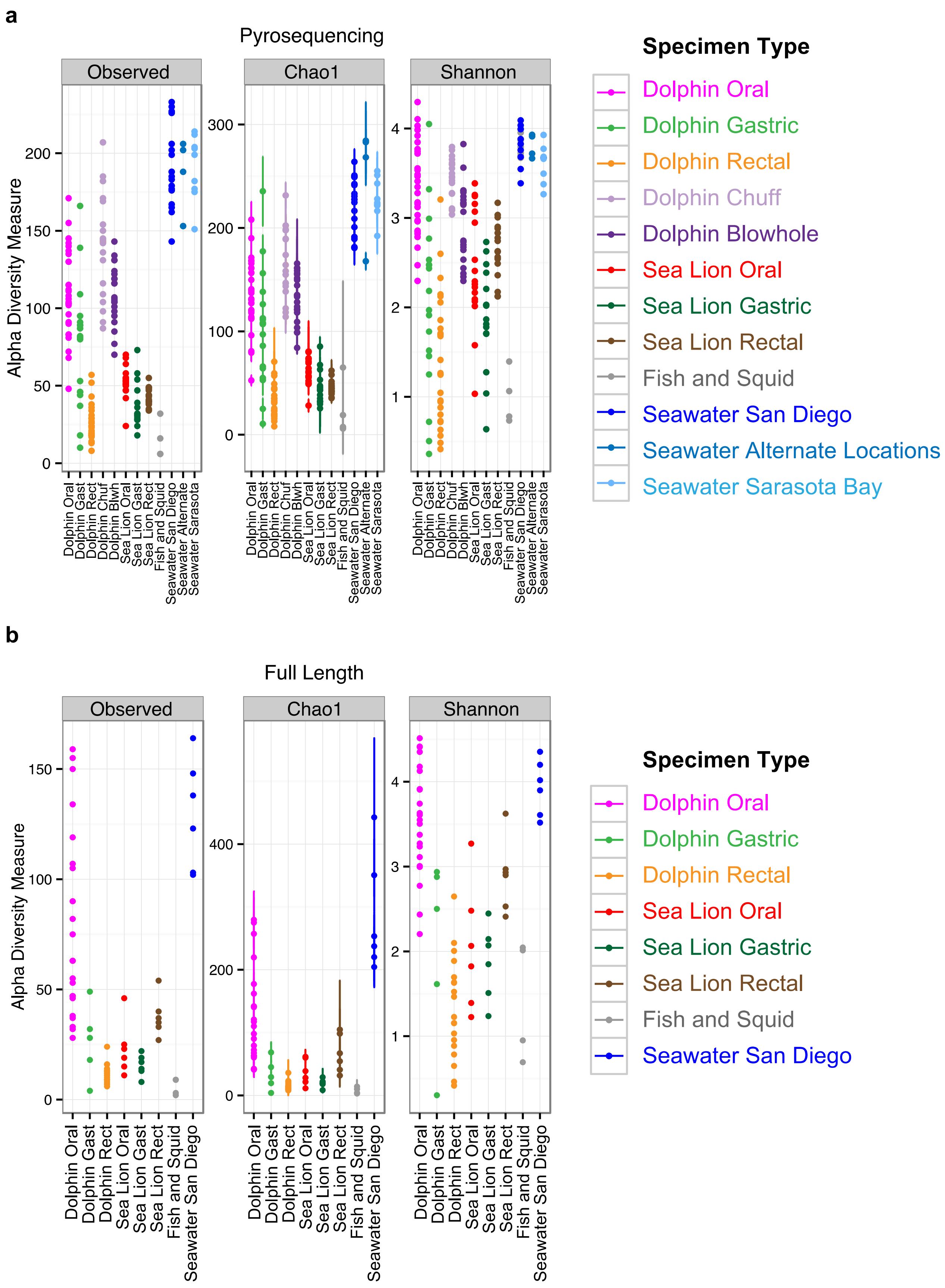

Supplementary Figure 5. Alpha diversity of the marine mammal microbiota found in pyrosequencing dataset (a) or near full length sequencing dataset (b). Graphs displays the total number of observed species (Observed) in each specimen type, the Chao1 index (an estimator for the total number of OTUs in each specimen if more sequencing were performed), and the Shannon diversity index. Colors indicate specimen type, while shapes indicate the location of the animal at the time of sampling. Only one timepoint per animal per specimen type is shown. Plots were generated in the R package phyloseq[2]; the R code is available in Supplementary Software 1 and 4. **a.** Pyrosequencing dataset. Only specimens with 372 or more reads (199 specimens), and only OTUs present at least 5 times in at least 2 specimens were included (n=1105). **b.** Near-full length dataset. All 77 specimens included here yielded 92 sequences or more (average: 260.1 reads). Note that no respiratory specimens were analyzed with this sequencing technique.

Supplementary Figure 6

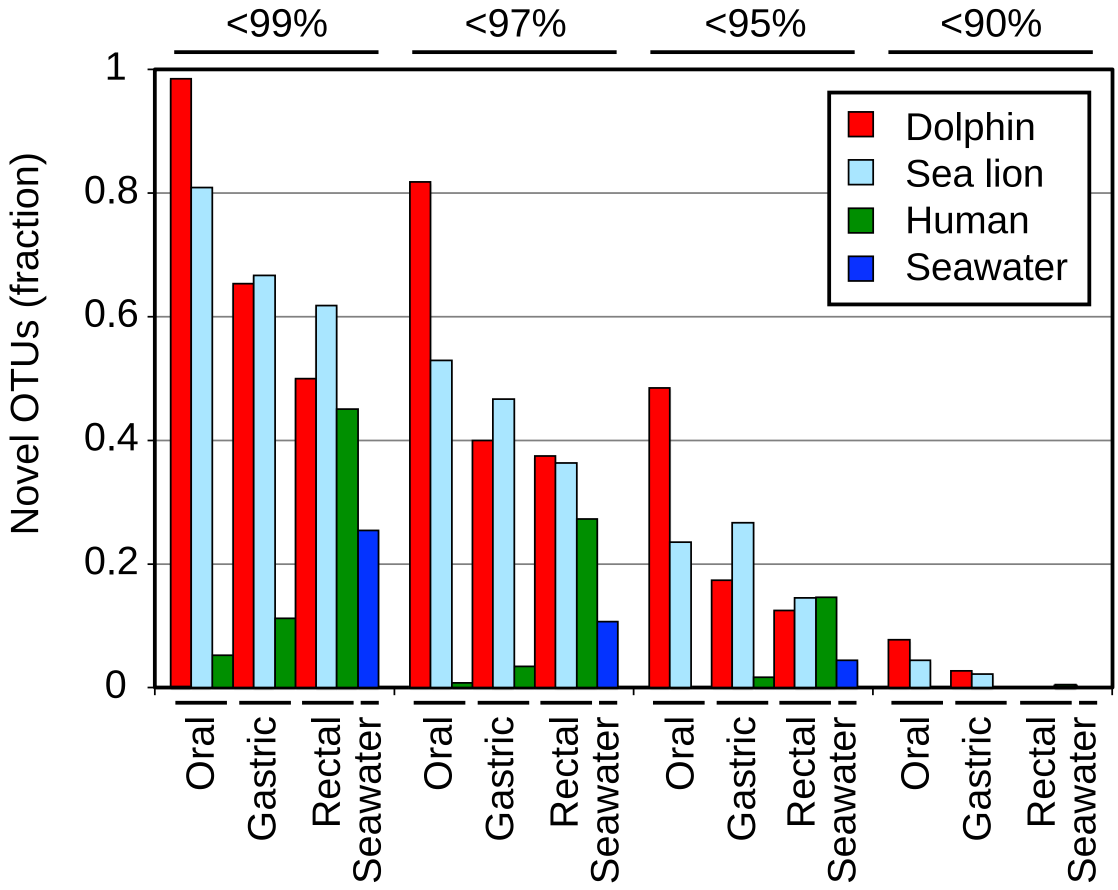

Supplementary Figure 6. Novel OTUs found in marine mammal microbiotas. FL sequence datasets were organized per host and per anatomical site, and rarefied to 1013 sequences each. To represent similarly constructed datasets that were considered novel at their original time of publication, sequences were added from a human oral study[3], a study on the human gastric microbiota[4], and from human rectum and stool[5]. Representative OTU sequences from seawater, dolphins, and sea lions were compared to NCBI's nucleotide database using a BLAST[6] search. For the three human studies, BLAST results were used from their year of publication. The fraction of OTU representatives without closely related published sequences is shown at four different percentage identity cutoffs. Details on rarefaction and BLAST search are provided in the Methods.

Supplementary Figure 7

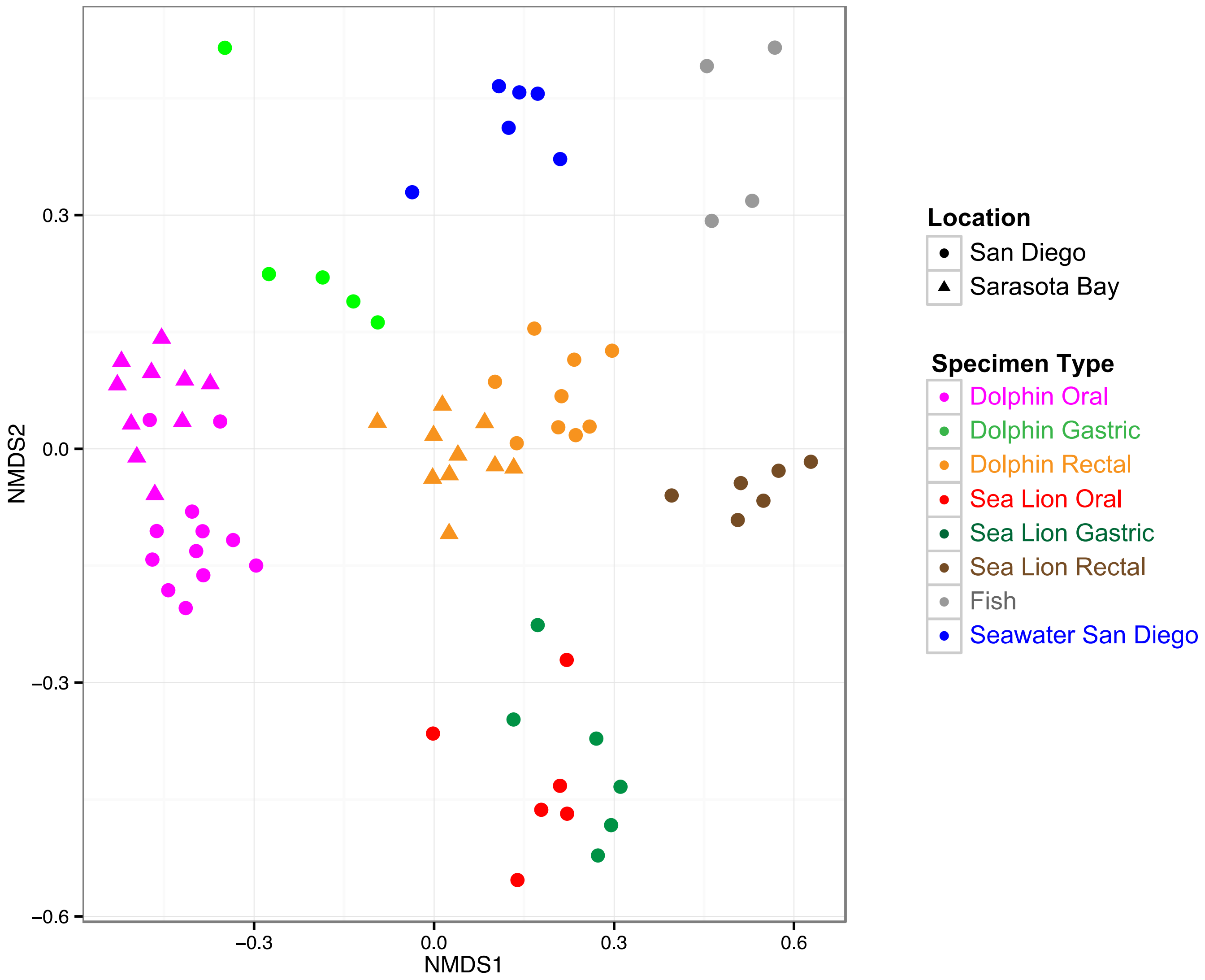

Supplementary Figure 7. NMDS Bray Curtis ordination of microbial communities analyzed in this study by near-full length sequencing. The same specimens are shown here as in Supplementary Figure 5b, except for 1 capelin and both squid specimens, because these shared too few FL-OTUs with the other specimens to be included. Datapoints are colored to specimen type, and shaped according to location. Note that no respiratory specimens were analyzed with this sequencing technique. The R code used to generate this plot is given in Supplementary Software 1.

Supplementary Figure 8

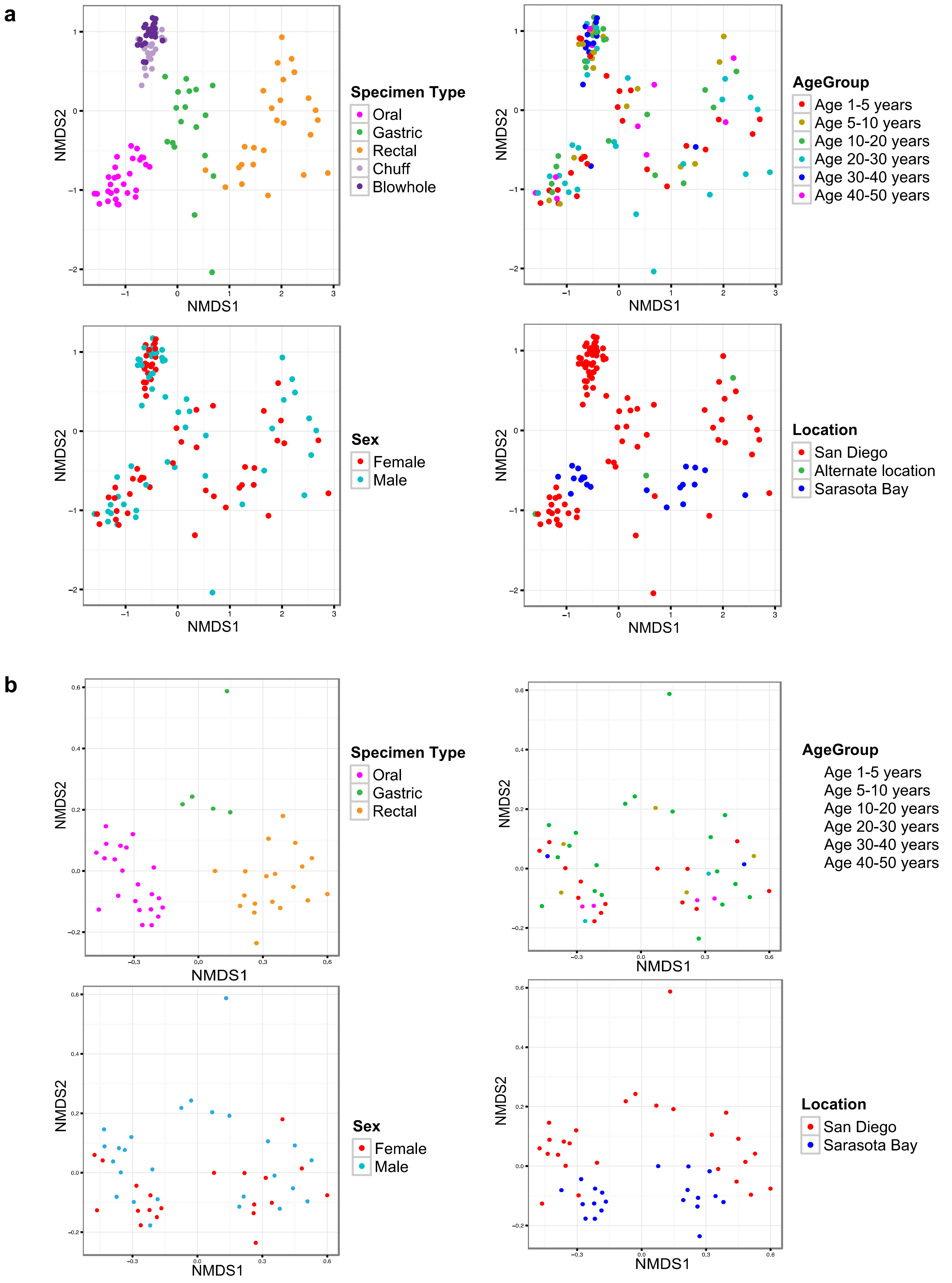

Supplementary Figure 8. NMDS Bray Curtis ordination of dolphin-associated microbial communities analyzed by pyrosequencing (a) or near-full length sequencing (b). For each sequencing technique, the same ordination plot is shown four times, but data points are colored according to body site (top left), animal age (top right), sex (bottom left) or location (bottom right). Only one timepoint per individual animal is shown. Plots were generated in the R package phyloseq[2]; the R code is available in Supplementary Software 1 and 4.

Supplementary Figure 9

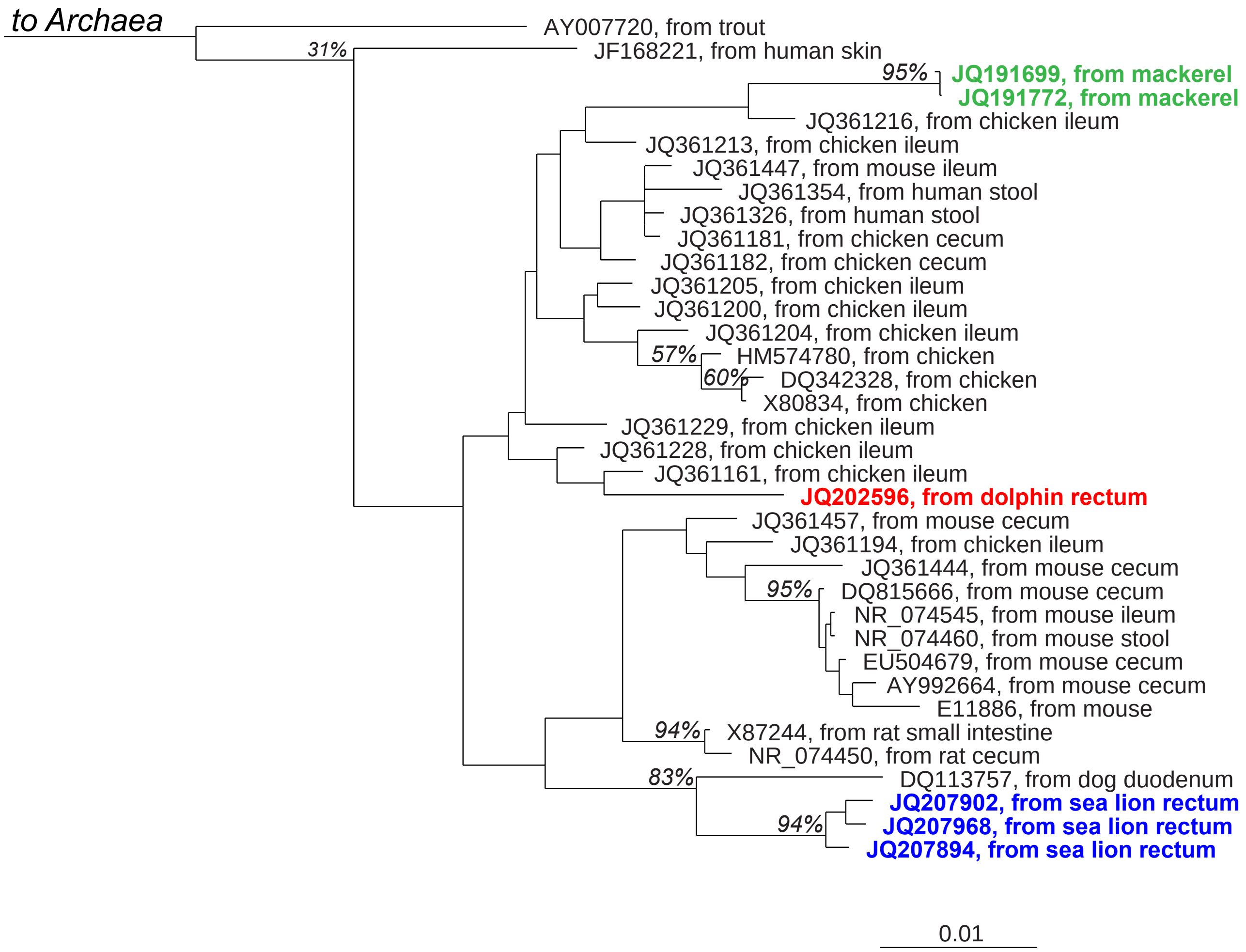

Supplementary Figure 9. Host specificity of *Arthromitus* (Segmented Filamentous Bacteria) sequences. *Arthromitus* sequences were obtained from ref.[7], from Genbank, and from this study. Dolphin (in red), sea lion (in blue), and mackerel (in green) sequences shown here are derived from FL sequences obtained in this study. The tree was generated in ARB[1] using a 558 nt mask and a neighbor joining method with 1000 bootstrap iterations. Only bootstrap values over 50% are shown.

Supplementary Figure 10

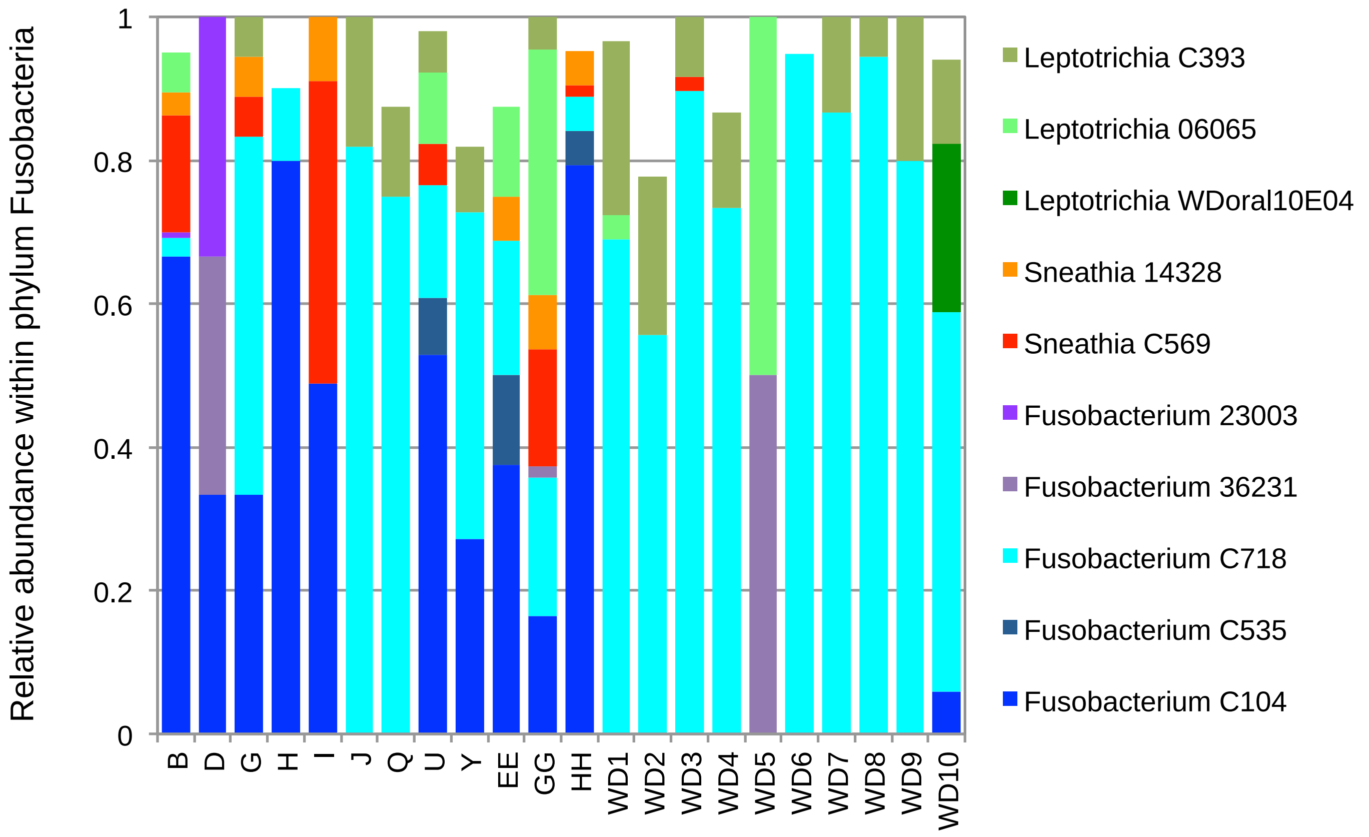

Supplementary Figure 10. Relative abundance of OTUs within phylum Fusobacteria found in the oral microbiota of MMP and wild dolphins, based on FL sequences. Dolphin oral specimens appear to often have one dominant Fusobacterium phylum species, usually *Fusobacterium* spp. OTU_C104 or OTU_C718.

Supplementary Figure 11

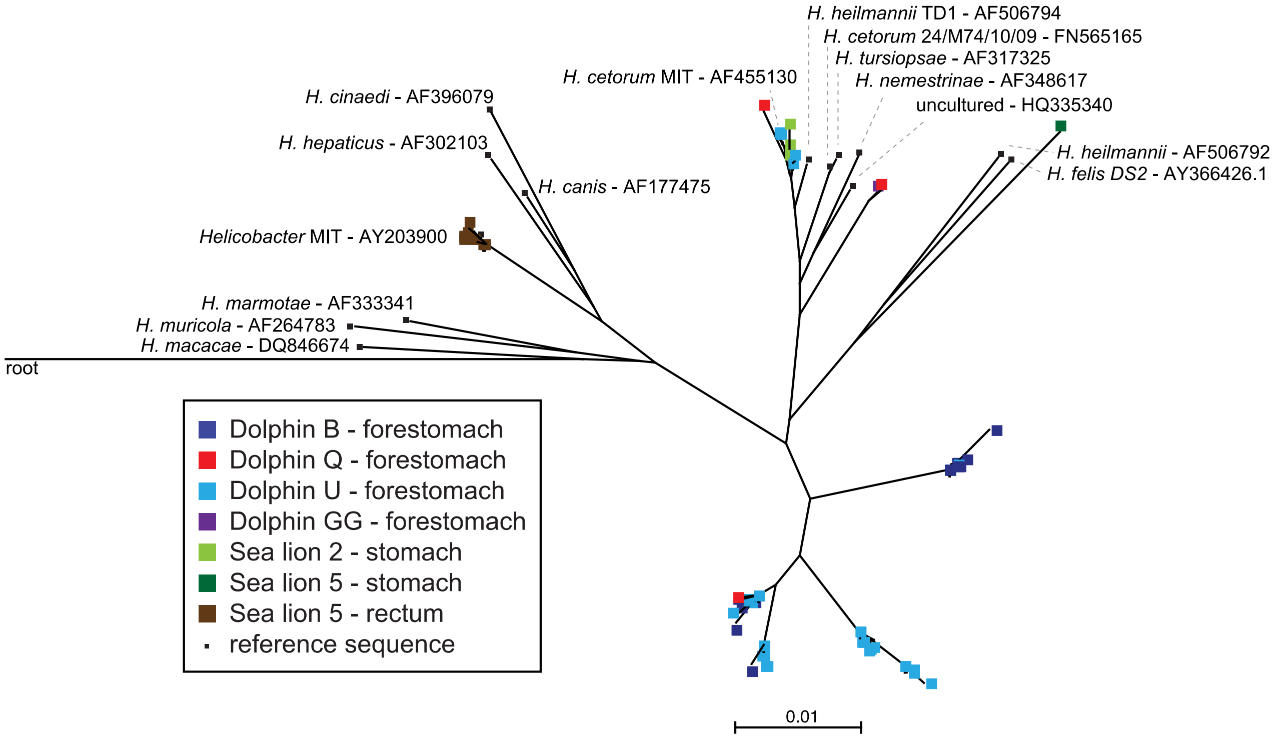

Supplementary Figure 11. Radial phylogenetic tree with *Helicobacter* sequences found in this study. The colored squares represent FL sequences obtained in this study. The small black squares represent published sequences; their Genbank accession number is shown. This tree was created in ARB[1] using a 1256 nt mask and a neighbor-joining method, and visualized using Dendroscope[8].

Supplementary Figure 12

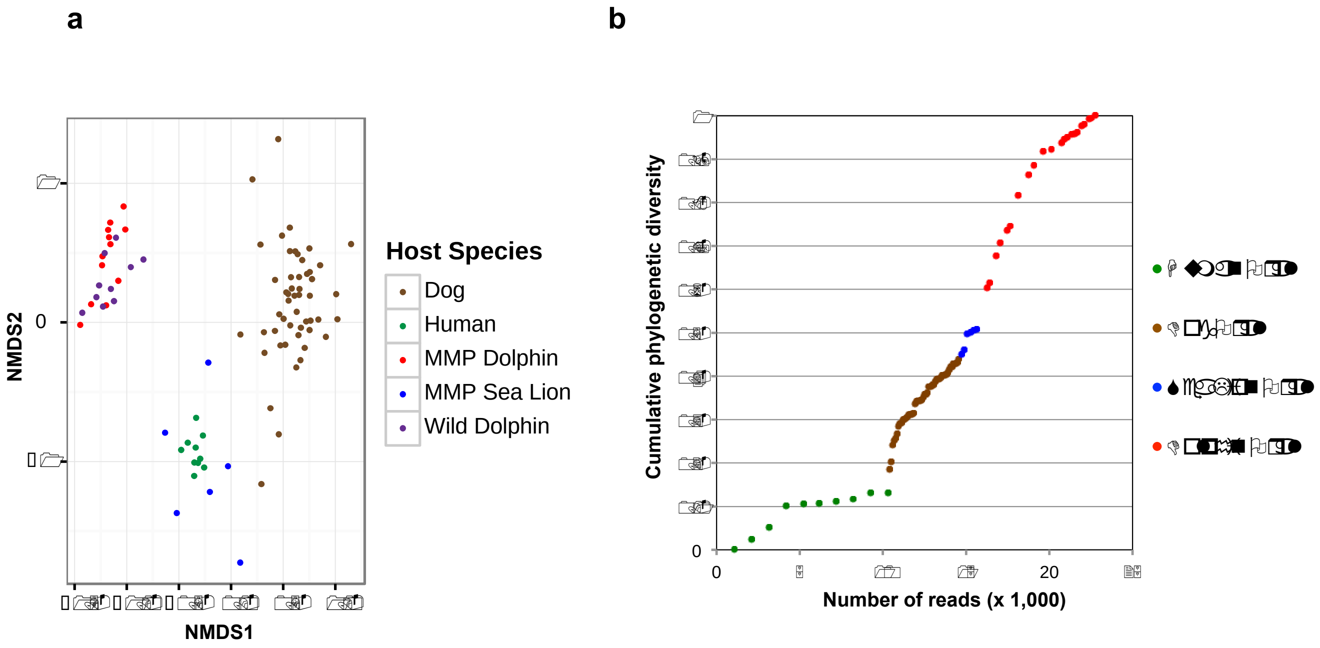

Supplementary Figure 12. Comparison of oral communities obtained from humans, dogs, dolphins and sea lions. a. NMDS ordination of Bray-Curtis distance analysis of FL oral bacterial communities found in marine mammals (this study), humans[3], and dogs[9]. **b.** Cumulative gain in phylogenetic diversity in oral specimen sequences from different hosts (same as shown in a). The same FL sequences as shown in **a** were used to calculate the phylogenetic diversity gain[10], i.e., a measurement of the increase in phylogenetic diversity (PD) upon addition of a new sample to a tree with sequences from other samples. The gain in PD was calculated in QIIME[11] using the beta_diversity.py script with the unifrac_g_full_tree metric, and an OTU table with the cumulative number of reads per OTU per specimen. The R code used to generate this plot is given in Supplementary Software 2.

Supplementary Tables

Supplementary Table 1. Clinical characteristics of animals included in this study. Specimens from a total of 48 bottlenose dolphins (38 MMP and 10 wild ranging animals) and 18 sea lions (all MMP) were included in this study. All animals were considered healthy at the time of sampling. The table shows relevant clinical data from each animal, which specimen types were collected, whether specimens were analyzed using clone library construction and Sanger sequencing, or by pyrosequencing, or both, as well as which animals were sampled multiple times. An asterisk in the sampling frequency column indicates that the respiratory specimens (chuff and blowhole) were obtained 3-4 years after collection of the oral, gastric, and rectal specimens. At the time of sampling, dolphin M and sea lions 9, 14, and 15 were located at a different alternate location than sea lion 20. Abbreviations: MMP, Marine Mammal Program San Diego; WD, wild free-ranging dolphin from Sarasota Bay; M, male, F, female; yrs, years.

**Bottlenose dolphins (*Tursiops truncatus*) N=48**						**Oral**	**Gastric**	**Rectal**	**Water**	**Oral**	**Gastric**	**Rectal**	**Chuff**	**Blowhole**	**Water**
**Animal**	**Location**	**Sex**	**Age (yrs)**	**Other Remarks**	**Sampling frequency**	Clone Library Samples				Pyrosequencing Samples					
B	MMP San Diego	M	27.6		One timepoint	●	●	●		●	●	●			
BBB	MMP San Diego	F	6.9		One timepoint								●	●	●
CC	MMP San Diego	M	6.1		One timepoint					●	●	●			
CCC	MMP San Diego	F	27.8		One timepoint									●	
D	MMP San Diego	F	24.1		One timepoint	●		●	●	●	●	●			
DD	MMP San Diego	M	13.8		One timepoint								●	●	●
EE	MMP San Diego	M	15.5		One timepoint *	●	●	●		●	●	●	●	●	●
EEE	MMP San Diego	F	24.3		One timepoint								●	●	●
FF	MMP San Diego	F	34.8		One timepoint								●	●	●
FFF	MMP San Diego	M	14.0		One timepoint								●	●	●
G	MMP San Diego	M	2.4		One timepoint	●			●	●	●	●			
GG	MMP San Diego	M	26.6		One timepoint	●	●								
GGG	MMP San Diego	M	11.0		One timepoint									●	●
H	MMP San Diego	F	3.8	Dietary indiscretion (eating seaweed/invertebrates)	Monthly	●		●	●	●	●	●			●
HH	MMP San Diego	M	25.4		One timepoint	●		●							

Supplementary Table 1 *(continued)*

**Bottlenose dolphins (*Tursiops truncatus*) N=48 (*continued*)**						**Oral**	**Gastric**	**Rectal**	**Water**	**Oral**	**Gastric**	**Rectal**	**Chuff**	**Blowhole**	**Water**
**Animal**	**Location**	**Sex**	**Age (yrs)**	**Other Remarks**	**Sampling frequency**	Clone Library Samples				Pyrosequencing Samples					
I	MMP San Diego	M	2.1		One timepoint	●			●	●	●	●			
J	MMP San Diego	M	4.1		Monthly	●		●		●	●	●			
JJ	MMP San Diego	F	2.9		One timepoint					●	●	●			
JJJ	MMP San Diego	F	10.3		One timepoint								●	●	
LLL	MMP San Diego	M	9.8		One timepoint								●	●	
M	MMP Alternate A	M	42.9		Monthly (6 months)					●	●	●			●
MMM	MMP San Diego	F	30.3		One timepoint									●	
N	MMP San Diego	F	15.1		One timepoint					●	●	●			
NNN	MMP San Diego	M	2.3		One timepoint								●	●	●
P	MMP San Diego	M	16.1		One timepoint *					●	●	●	●	●	●
Q	MMP San Diego	M	26.9		Monthly	●	●	●		●	●	●			●
QQ	MMP San Diego	M	3.6		One timepoint								●	●	
RR	MMP San Diego	M	7.7		One timepoint								●	●	
S	MMP San Diego	F	25.9	Lactating dam	Monthly *					●	●	●	●	●	●
U	MMP San Diego	M	28.7		Monthly *	●	●	●	●	●	●	●	●	●	●
V	MMP San Diego	F	47.1		One timepoint *					●	●	●	●	●	●
WW	MMP San Diego	F	34.2		One timepoint								●	●	●
X	MMP San Diego	F	6.0	Lactating dam	One timepoint					●	●	●			
XX	MMP San Diego	F	30.5		One timepoint								●	●	●
Y	MMP San Diego	F	43.0	Lactating dam	Monthly	●		●	●	●	●	●			●
YY	MMP San Diego	F	32.5	Skin lesions	One timepoint								●	●	●
Z	MMP San Diego	F	29.5	Lactating dam, mother of QQ	One timepoint					●		●			
ZZ	MMP San Diego	M	9.3		One timepoint								●	●	
WD01	Sarasota Bay	F	8.5		One timepoint	●		●		●		●			●
WD02	Sarasota Bay	F	5		One timepoint	●		●		●		●			●
WD03	Sarasota Bay	F	10	Many skin rakes	One timepoint	●		●		●		●			●
WD04	Sarasota Bay	F	2	Calf of dolphin WD07	One timepoint	●		●		●		●			●
WD05	Sarasota Bay	M	17		One timepoint	●		●		●		●			●
WD06	Sarasota Bay	M	21		One timepoint	●		●		●		●			●
WD07	Sarasota Bay	F	39	Mother of calf WD04	One timepoint	●		●		●		●			●
WD08	Sarasota Bay	F	26	Mother of calf WD9	One timepoint	●		●		●		●			●
WD09	Sarasota Bay	M	2	Calf of WD8	One timepoint	●		●		●		●			●
WD10	Sarasota Bay	F	5	Re-sampling of previously rehabilitated dolphin	One timepoint	●		●		●		●			●

Supplementary Table 1 *(continued)*

**California sea lions (*Zalophus californianus*) N=18**						**Oral**	**Gastric**	**Rectal**	**Oral**	**Gastric**	**Rectal**	**Water**
**Animal**	**Location**	**Sex**	**Age (yrs)**	**Other Remarks**	**Sampling frequency**	Clone Library Samples			Pyrosequencing Samples			
Sea Lion 2	MMP San Diego	M	3.6	Dental - endodontal disease	One timepoint	•	•	•	•	•	•	•
Sea Lion 4	MMP San Diego	M	7.6	Dental - endodontal disease	One timepoint	•	•	•	•	•	•	
Sea Lion 5	MMP San Diego	M	3.7		One timepoint	•	•	•	•	•	•	
Sea Lion 6	MMP San Diego	M	27.7		One timepoint	•	•	•	•	•	•	
Sea Lion 7	MMP San Diego	M	26.7		One timepoint	•	•	•	•	•	•	
Sea Lion 8	MMP San Diego	M	1.7		One timepoint	•	•	•	•	•	•	
Sea Lion 9	MMP Alternate A	M	8.8		One timepoint				•	•	•	•
Sea Lion 10	MMP San Diego	M	25.8		One timepoint				•	•	•	
Sea Lion 11	MMP San Diego	M	1.8		One timepoint				•	•	•	
Sea Lion 12	MMP San Diego	M	7.9		One timepoint				•	•	•	
Sea Lion 13	MMP San Diego	M	7.9		One timepoint				•	•	•	
Sea Lion 14	MMP Alternate A	M	2.4		One timepoint				•	•	•	•
Sea Lion 15	MMP Alternate A	M	9.4	Dental - endodontal disease	One timepoint				•	•	•	•
Sea Lion 16	MMP San Diego	M	2.6		One timepoint				•	•	•	
Sea Lion 17	MMP San Diego	M	2.5		One timepoint				•	•	•	
Sea Lion 18	MMP San Diego	M	2.7		One timepoint				•	•	•	
Sea Lion 19	MMP San Diego	M	27.5		One timepoint				•		•	
Sea Lion 20	MMP Alternate B	M	2.3		One timepoint				•	•	•	•

Supplementary Table 2. Microbial gut communities from other mammals that were most closely related to microbiotas from dolphins and sea lions. Distances between gut communities obtained from dolphins (left) or sea lions (right), and gut communities from other mammals (references included) were calculated using Bray-Curtis (ordination shown in Fig. 7). The lowest values (microbiotas most closely related to those of dolphins and sea lions, respectively) are shown here. PD, phylogenetic diversity.

Animal species (abbreviation, study)	Averaged PD to gut community of dolphins	Animal species (abbreviation, study)	Averaged PD to gut community of sea lions
Bottlenose dolphins, averaged (this study)	0.5207	Hooded Seal (HdSeal, ref.[13])	0.4571
Polar Bear 1 (PB1, ref.[12])	0.6435	Average Sealion (this study)	0.4598
Polar Bear 2 (PB2, ref.[12])	0.6615	Grey Seal (GrSeal, ref.[13])	0.5768
Black Bear (BB1, ref.[12])	0.6837	Wolf (wolf, ref.[17])	0.6464
Red Panda (RPSD, ref.[12])	0.7104	Harbor Seal (HbSeal, ref.[13])	0.6593
Hooded Seal (HdSeal, ref.[13])	0.7472	Dog (dog, ref.[16])	0.6810
Black Bear (BB2, ref.[12])	0.7477	Bushdog (bdog3, ref.[12])	0.6813
Hedgehog (HH, ref.[12])	0.7514	Bushdog (bdog1, ref.[12])	0.7102
Yangtze Finless Porpoise (YFPorp, ref.[14])	0.7588	Hyena (HY1, ref.[12])	0.7199
Lion (LI1, ref.[12])	0.7671	Hyena (HY2, ref.[12])	0.7227
Sea lions, averaged (this study)	0.7679	Armadillo (arma, ref.[12])	0.7406
Lion (LI2, ref.[12])	0.7715	Cheetah (CE3, ref.[12])	0.7608
Red Panda (RP, ref.[12])	0.7834	Human (HumDethC1, ref.[18])	0.7614
Cheetah (CE2, ref.[12])	0.7928	Human (HumDethB1, ref.[18])	0.7623
Bushdog (bdog3, ref.[12])	0.7941	Geoffreys Marmoset (MAR, ref.[12])	0.7659
Polar Bear (PolBear, ref.[15])	0.8018	Bottlenose dolphins, average (this study)	0.7679
Dog (dog, ref.[16])	0.8026	Human (HumDethA1, ref.[18])	0.7716
Wolf (wolf, ref.[17])	0.8044	Lion (LI3, ref.[12])	0.7752

Supplementary Table 3. Primers and probes used for the quantitative PCRs. These primers and probes were used (results shown in Fig. 3C) to detect specific bacterial groups that were found to be abundant in a particular specimen type. All primers and probe sequences are shown 5' to 3'. F, forward primer; R, reversed primer; P, Taqman probe.

Target taxon	Abundant anatomical site	Clone ID	Genbank Accession Number	PCR product size (bp)	Forward primer (5' to 3')
Actinobacillus delphinicola/scotia	Dolphin rectum	B031	JQ204253	327	F: GGG AAT CTG TTT YAT GGA GG<br>R: TCC TCA CCA CCG AAA GAA C<br>P: FAM-TT TAA TCT CTC GAT ATT ACG CGG TAT TAG-TAMRA
Tenericutes spp.	Dolphin stomach	A476	JQ194483	336	F: CTA ATA CCG AAT AAG CTC TTG G<br>R: AAA TGG TAC AGT CAA ATA AAG AAC<br>P: FAM-CC AAA GGA TCG CCT TTA GAT GA-TAMRA
Fusobacterium spp.	Dolphin mouth	C104	JQ216612	364	F: TAG AGA TAT ATC AGT GCT TCT TCG<br>R: CGC AAC ATT GCT GAT TTG<br>P: FAM-CA CCC TCG CAG GTT CGC AA-TAMRA
Pelagibacter ubique	Seawater	O614	JQ196291	274	F: TAC CGG ATA AGT CTT TAC GG<br>R: AGT CAT TTT CTT CCC CGA C<br>P: FAM-AG GCT CAT CCA ATG GTG CAT AAA- TAMRA

Supplementary References

1. Ludwig, W. *et al*. ARB: a software environment for sequence data. Nucleic Acids Res 32, 1363-1371, doi:10.1093/nar/gkh29332/4/1363 (2004).
2. McMurdie, P. J. & Holmes, S. phyloseq: An R Package for Reproducible Interactive Analysis and Graphics of Microbiome Census Data. PLOS One 8, doi:10.1371/journal.pone.0061217 (2013).
3. Bik, E. M. *et al.* Bacterial diversity in the oral cavity of 10 healthy individuals. ISME J 4, 962-974, doi:10.1038/ismej.2010.30 (2010).
4. Bik, E. M. *et al.* Molecular analysis of the bacterial microbiota in the human stomach. Proc Natl Acad Sci U S A 103, 732-737, doi:10.1073/pnas.0506655103 (2006).
5. Eckburg, P. B. *et al.* Diversity of the human intestinal microbial flora. Science 308, 1635-1638, doi:10.1126/science.1110591 (2005).
6. Altschul, S. F., Gish, W., Miller, W., Myers, E. W. & Lipman, D. J. Basic local alignment search tool. Journal of molecular biology 215, 403-410, doi:10.1016/S0022-2836(05)80360-2 (1990).
7. Yin, Y. S. *et al*. Comparative analysis of the distribution of segmented filamentous bacteria in humans, mice and chickens. ISME Journal 7, 615-621, doi:10.1038/Ismej.2012.128 (2013).
8. Huson, D. H.& and Scornavacca, C. Dendroscope 3: An interactive tool for rooted phylogenetic trees and networks, Systematic Biology , doi: 10.1093/sysbio/sys062 (2012).
9. Dewhirst, F. E. et al. The canine oral microbiome. PLOS ONE 7, e36067, doi:10.1371/journal.pone.0036067 (2012).
10. Faith, D. P. Conservation evaluation and phylogenetic diversity. Biological Conservation 61, 1-10, doi:10.1016/0006-3207(92)91201-3 (1992).
11. Caporaso, J. G. et al. QIIME allows analysis of high-throughput community sequencing data. Nat Methods 7, 335-336, doi:10.1038/nmeth.f.303 (2010).
12. Ley, R. E. et al. Evolution of mammals and their gut microbes. Science 320, 1647-1651, doi:10.1126/science.1155725 (2008).
13. Glad, T. et al. Ecological characterisation of the colonic microbiota in arctic and sub-arctic seals. Microb Ecol 60, 320-330, doi:10.1007/s00248-010-9690-x (2010).

14. McLaughlin, R. W., Chen, M. M., Zheng, J. S., Zhao, Q. Z. & Wang, D. Analysis of the bacterial diversity in the fecal material of the endangered Yangtze finless porpoise, *Neophocaena phocaenoides asiaeorientalis*. Mol Biol Rep 39, 5669-5676, doi:10.1007/S11033-011-1375-0 (2012).
15. Glad, T. et al. Bacterial diversity in faeces from polar bear (*Ursus maritimus*) in Arctic Svalbard. BMC Microbiol 10, 10, doi:10.1186/1471-2180-10-10 (2010).
16. Suchodolski, J. S., Camacho, J. & Steiner, J. M. Analysis of bacterial diversity in the canine duodenum, jejunum, ileum, and colon by comparative 16S rRNA gene analysis. FEMS Microbiology Ecology 66, 567-578, doi:10.1111/J.1574-6941.2008.00521.X (2008).
17. Zhang, H. H. & Chen, L. Phylogenetic analysis of 16S rRNA gene sequences reveals distal gut bacterial diversity in wild wolves (*Canis lupus*). Mol Biol Rep 37, 4013-4022, doi:10.1007/S11033-010-0060-Z (2010).
18. Dethlefsen, L., Huse, S., Sogin, M. L. & Relman, D. A. The Pervasive Effects of an Antibiotic on the Human Gut Microbiota, as Revealed by Deep 16S rRNA Sequencing. PLoS Biol 6, e280, doi:10.1371/journal.pbio.0060280 (2008).
