# Supplementary material for: Marine mammals harbor unique microbiotas shaped by and yet distinct from the sea
Source: Nat Commun. 2016 Feb 3;7:10516. doi: 10.1038/ncomms10516 (PMC4742810; doi:10.1038/ncomms10516)
Supplement: Supplementary Software 1 — R code for analysis of FL sequences obtained from dolphins, sea lions, fish food, and seawater. [file ncomms10516-s2.docx]

**Bik et al., Supplementary Software 1**

**R code for analysis of FL sequences obtained from dolphins, sea lions, fish food, and seawater.**

**Author: Elisabeth Bik, Stanford University - November 2015**

**========================================================**

# R markup file with analyses done on a full length dataset from dolphin and sea lion specimens: oral, gastric, and rectal specimens, as well as seawater collected next to the animals, and fish and squid samples used to feed the animals. Dolphins were from 2 locations: MMP in San Diego, and Sarasota Bay (wild dolphins). All sea lions were located at MMP. All specimens here were single timepoints.

# Samples were extracted with the QIAamp DNA extraction kit, tissue protocol. Datasets were created by amplification of DNA with primers 8FM/B forward and 1391R reversed. PCR products were cloned and sequenced from both ends, and assembled using Sequencher. In case the PCR gave too much (host?) background, samples were amplified using 8FM/B forward and 806 reversed, generating a shorter fragment (but still longer than the pyrosequencing dataset).

# Sequences aligned to the Greengenes alignment and imported into ARB. They were manually checked for chimeras, assigned to OTUs, and compared to reference sequences, all within ARB. OTU representatives were additionally screened for chimeras using Mallard.

**========================================================**

**#### Libraries and initialization:**

```{r}

R.Version()

library("phyloseq")

packageVersion("phyloseq")

library("ggplot2")

packageVersion("ggplot2")

library("ape")

packageVersion("ape")

library("DESeq2")

packageVersion("DESeq2")

library(plotrix)

packageVersion("plotrix")

# source("http://bioconductor.org/biocLite.R")

# biocLite("vsn")

library("plyr")

packageVersion("plyr")

library("doParallel")

packageVersion("doParallel")

library("foreach")

packageVersion("foreach")

theme_set(theme_bw())

```

# Choose your workspace (change to your own environment)

# setwd("/Users/elies/Desktop/")

**#### Creating a new phyloseq (can skip and import once done)**

```{r}

# Import OTU table in biom format

OTUtable <- import_biom("FL_OTUtable.biom")

colnames(tax_table(OTUtable)) <- c("Kingdom", "Phylum", "Class", "Order", "Family", "Genus", "Species")

# Import a mapping file in Qiime format

mapfile <- import_qiime(mapfilename="FL_Map.txt")

# Importing a tree

treefile <- import_qiime(treefilename="FL_OTUreps.tre.txt")

# create a single phyloseq object with a short name

ps <- merge_phyloseq(OTUtable, mapfile, treefile)

print(ps)

# Look at distribution of the number of samples in which each taxa is observed:

otab <- as(otu_table(ps), "matrix") # Taxa are rows

present_absent <- (otab > 0)

nsamples <- apply(present_absent, 1, sum)

hist(log(nsamples), 60)

**#### Plot tree, alpha and beta diversity**

```{r}

print(ps)

# plot tree

plot_tree(ps, color = "SampleTypeSimple", justify = "left", size = "Abundance")

# ordination on single timepoint set: Bray Curtis / NMDS (Supplementary Figure 7).

# I got errors when I included FishCapln2 and Sqd1 and 2; when I excluded these three it went fine.

# So I took these out of the analysis (too little overlap with any of the other samples)

ord_NMDS_bray = ordinate(ps, "NMDS", "bray")

p <- plot_ordination(ps, ord_NMDS_bray, color="SampleTypeSimple", shape="Location",title="NMDS Bray Curtis Full Length")

p

p = p + aes(size=2) + guides(size=FALSE)

p

# Creating and exporting the distance matrix (Bray Curtis)

distbray = phyloseq::distance(ps, method="bray", type="samples")

distb = as.matrix(distbray)

distb[upper.tri(distb)] = NA

distb

write.table(distb, "DistanceMatrix_DolSL_Bray_July2015.txt", sep="\t", col.names=NA, quote=F)

# make a subset containing only dolphin samples, single tp over 200 reads

dol <- subset_samples(ps, AnimalSpecies=="Dol")

print(dol)

# ordination of dolphin samples on sample type, age, sex, location (Supplementary Figure 8b).

ord_NMDS_bray_dol = ordinate(dol, "NMDS", "bray")

pSampleType <- plot_ordination(dol, ord_NMDS_bray_dol, color="SampleTypeSimple", title="NMDS Bray Curtis Dolphin FL") + aes(size=2) + guides(size=FALSE)

pSampleType

pSex <- plot_ordination(dol, ord_NMDS_bray_dol, color="Sex", title="NMDS Bray Curtis Dolphin FL") + aes(size=2) + guides(size=FALSE)

pSex

pAge <- plot_ordination(dol, ord_NMDS_bray_dol, color="AgeGroup", title="NMDS Bray Curtis Dolphin FL") + aes(size=2) + guides(size=FALSE)

pAge

pLoc <- plot_ordination(dol, ord_NMDS_bray_dol, color="Location", title="NMDS Bray Curtis Dolphin FL") + aes(size=2) + guides(size=FALSE)

pLoc

# Plot basic alpha diversity measures (Sobs, Chao1, Shannon), facetted per method, grouped/colored per SampleTypeSimple (Supplementary Figure 5b).

plot_richness(ps, x="SampleTypeSimple", color="SampleTypeSimple", measures=c("Observed", "Chao1", "Shannon"), title="Full Data")

```
