# Supplementary material for: Marine mammals harbor unique microbiotas shaped by and yet distinct from the sea
Source: Nat Commun. 2016 Feb 3;7:10516. doi: 10.1038/ncomms10516 (PMC4742810; doi:10.1038/ncomms10516)
Supplement: Supplementary Software 2 — R code for comparison of FL oral sequences obtained in our study to those from humans and dogs. [file ncomms10516-s3.docx]

**Bik et al., Supplementary Software 2**

**R code for comparison of FL oral sequences obtained in our study to those from humans and dogs**

**Author: Elisabeth Bik, Stanford University, November 2015**

**================================================================**

# Here we will compare cloned 16S rRNA sequences (near full length, FL) from oral samples from dolphins and sea lions obtained in our study, to those from oral samples from humans and dogs.

# Our sequences were generated by 8Fx1391R PCR (1400 bps), from MMP and wild dolphins, and MMP sea lions.

# Reference sequences were used from the following 2 studies:

# 1. Bik et al. 2010 Bacterial diversity in the oral cavity of 10 healthy individuals. ISME J. 2010 Aug;4(8):962-74 (generated by our lab)

# 2. Dewhirst et al. 2012. The canine oral microbiome. PLoS ONE: e36067

**================================================================**

**#### Initializing libraries and working directory**

```{r}

# Load the libraries that are needed

library("phyloseq")

packageVersion("phyloseq")

library("ggplot2")

packageVersion("ggplot2")

theme_set(theme_bw())

library("plyr")

# set the working directory

setwd("/Users/elies 1/Desktop/") # change to your own directory

```

**#### Merging the data into a phyloseq object**

```{r}

# Generate a new Phyloseq object with oral samples from 4 species

# contains seqeuences from humans (Bik 2010), dogs (Dewhirst 2012), dolphins, sea lions (our study)

# import otu table (biom file)

OTUtable <- import_biom("Oral_4species_OTUtable_Dec2014.biom")

# import mapping file

mapfile <- import_qiime(mapfilename="Oral_DL_WD_SL_Hum_dog_Mapping_Dec2014.txt")

# import a tree

treefile <- import_qiime(treefilename="Oral_DL_SL_Hum_Dog_Dec2014.tre")

# create a single phyloseq object

OralPS <- merge_phyloseq(OTUtable, mapfile, treefile)

print(OralPS)

# make histograms

hist(log(taxa_sums(OralPS)), 60)

otab <- as(otu_table(OralPS), "matrix") # Taxa are rows

present_absent <- (otab > 0)

nsamples <- apply(present_absent, 1, sum)

hist(log(nsamples), 60)

# saving the phyloseq object, so next time we can quickly import it

save(OralPS, file = "Oral_DL_SL_Hum_Dog.RData")

```

**#### loading the phyloseq object, once it has been created, making tree, plot ordination**

```{r}

# load the phyloseq object created above

load("Oral_DL_SL_Hum_Dog.RData")

print(OralPS)

# merge OTUs that are very similar, try h=0.05

# this is especially important for Bray Curtis analysis on datasets that were merged from different studies, hence little overlap between OTUs expected

mOralPS <- tip_glom(OralPS, h=0.05)

print(mOralPS)

hist(log(taxa_sums(mOralPS)), 60)

plot_tree(mOralPS, color = "AnimalSpecies", justify = "left", size = "Abundance", title="TipMerged h=0.05 - 495 taxa - 89 samples")

# Perform an ordination and then plot it. We went with the Bray Curtis NMDS plot (See supplementary Figure 12a).

ord_NMDS_bray = ordinate(mOralPS, "NMDS", "bray")

plot_ordination(mOralPS, ord_NMDS_bray, color="AnimalSpecies", title="NMDS Bray")

ord_PCoA_bray = ordinate(mOralPS, "PCoA", "bray")

plot_ordination(mOralPS, ord_PCoA_bray, color="AnimalSpecies", title="PCoA Bray")

ord_PCoA_unifrac = ordinate(mOralPS, "PCoA", "unifrac")

plot_ordination(mOralPS, ord_PCoA_unifrac, color="AnimalSpecies", title="PCoA Unifrac")

```
