# Supplementary material for: Marine mammals harbor unique microbiotas shaped by and yet distinct from the sea
Source: Nat Commun. 2016 Feb 3;7:10516. doi: 10.1038/ncomms10516 (PMC4742810; doi:10.1038/ncomms10516)
Supplement: Supplementary Software 3 — R code for comparison of distal gut FL sequences obtained from dolphins, sea lions, to those from published datasets. [file ncomms10516-s4.docx]

**Bik et al., Supplementary Software 3**

**R code for comparison of distal gut FL sequences obtained from dolphins, sea lions, to those from published datasets.**

title: "MammalComparison with dolphins, sealions, manatees"

authors: Susan Holmes, Ben Callahan, Elisabeth Bik – Stanford University

start date: Monday, September 28,2015

working date: Tuesday, November 17, 2015

generated using Rmd

**================================================================**

# R code used for the ordination of distal gut bacterial communities from marine and terrestrial mammals

# For marine mammals, we will use our own data (rectal communities from bottlenose dolphins, sea lions) supplemented with data from manatees (our lab, unpublished), and published data from river porpoises, seals, and a dugong.

# The terrestrial mammal data that we will use here is mainly derived from the Ruth Ley study (Science 2008) (chimeras removed), supplemented with data from gut communities from humans, dogs, mice, wolves, and bears. References and other details are given in Supplementary Data 4.

**================================================================**

Load and massage data:

================================================================

Load the data in phyloseq form:

```{r}

# You need to change the directory to yours

# Loading the phyloseq object created above

library(phyloseq)

#setwd("/Users/eliesbik/Desktop")

load("MammalGut_Oct2015.RData")

print(MammalGutOct2015)

```

```{r}

# Check if there are any OTUs in this dataset that have zero reads

# This is important when using publically available datasets

# There were not any - OK

sum(taxa_sums(MammalGutOct2015) == 0)

summary(sample_sums(MammalGutOct2015))

#Merge OTUs into genera based on taxonomy - will take 2 min

MammalGutGenusMerged = tax_glom(MammalGutOct2015, "Genus")

print(MammalGutGenusMerged)

summary(sample_sums(MammalGutGenusMerged))

otu.tab<-MammalGutGenusMerged@otu_table

write.table(t(otu.tab), "MammalGutGenusMerged_OTUtable.txt", sep="\t", quote=F, col.names=NA)

```

Inspect sample data:

```{r}

ps <- MammalGutGenusMerged

sample_variables(ps)

table(sample_data(ps)$HostSpeciesCommon)

table(sample_data(ps)$Provenance) #??

table(sample_data(ps)$Habitat)

table(sample_data(ps)$Diet)

table(sample_data(ps)$Diet, sample_data(ps)$Habitat)

table(sample_data(ps)$HostGroup)

table(sample_data(ps)$Diet, sample_data(ps)$HostGroup)

table(sample_data(ps)$HostOrder) # Will change the Artiodactyla to Cetartiodactyla in ms plots

```

Initial ordination

================================================================

```{r}

library(vegan)

library(ggplot2)

phys=MammalGutGenusMerged

physr=phys

DolphinFactor=factor(rep("NotDolphin",135),levels=c("NotDolphin","Dolphin"))

DolphinFactor[ which(sample_data(phys)[,3]=="AtlanticBottleNoseDolphin")]="Dolphin"

sample_data(physr)=data.frame(sample_data(phys),DolphinFactor)

dolphin.ca <- ordinate(physr, "CCA")

p1 <- plot_ordination(physr, dolphin.ca, type="split", color="Phylum", shape="DolphinFactor")

#, label=sample_names(psr))

my.cols <- rainbow(length(levels(p1$data$Phylum)))

names(my.cols) <- levels(p1$data$Phylum)

my.cols["samples"] <- "black"

p3 <- plot_ordination(physr, dolphin.ca, type="split", color="Phylum", shape="DolphinFactor", label="X.SampleID")

p3 + scale_colour_manual(values=my.cols)

```

Serious outlier: here it is documented before taking out:

```{r}

which(sample_data(physr)$X.SampleID=="bat")

dolphin.ca$CA$u[1:10,1:3]

which(abs(dolphin.ca$CA$v[,1])>10)

tax_table(physr)[c(29,147),]

```

Removed outlier bat

```{r}

phys=prune_samples(sample_data(physr)$X.SampleID!="bat", physr)

dolphin.ca <- ordinate(phys, "CCA")

p1 <- plot_ordination(phys, dolphin.ca, type="split", color="Phylum", shape="DolphinFactor")

#, label=sample_names(psr))

p3 <- plot_ordination(phys, dolphin.ca, type="split", color="Phylum", shape="DolphinFactor", label="X.SampleID")

p3 + scale_colour_manual(values=my.cols)

```

Same plots with and without the bat

================================================================

```{r}

###new data is physr with bat and phys without

dolphin.ccan <- ordinate(physr, "CCA",formula = otu_matrix ~ DietNumber + HabitatNumber + as.factor(HostNumber))

p3n <- plot_ordination(physr, dolphin.ccan, type="samples", shape="DolphinFactor", label="X.SampleID")

p3n + scale_colour_manual(values=my.cols) + ggtitle("With Bat")

##################without bat

dolphin.ccan <- ordinate(phys, "CCA",formula = otu_matrix ~ DietNumber +

HabitatNumber + as.factor(HostNumber))

p3n <- plot_ordination(phys, dolphin.ccan, type="samples", shape="DolphinFactor", label="X.SampleID")

p3n + scale_colour_manual(values=my.cols) + ggtitle("Without Bat")

```

Change the signs to make it look the same as original

-------------------------------------------------------

```{r}

dolphin.ccan$CCA$wa[,1]=(-dolphin.ccan$CCA$wa[,1])

#dolphin.ccan$CCA$wa[,2]=(-dolphin.ccan$CCA$wa[,2])

dolphin.ccan$CCA$v[,1]=(-dolphin.ccan$CCA$v[,1])

#dolphin.ccan$CCA$v[,2]=(-dolphin.ccan$CCA$v[,2])

p3n <- plot_ordination(phys, dolphin.ccan, type="samples", shape="DolphinFactor", label="X.SampleID")

p3n + scale_colour_manual(values=my.cols)

```

```{r}

dolphin.ccan <- ordinate(phys, "CCA",formula = otu_matrix ~ DietNumber + HabitatNumber)

p3n <- plot_ordination(phys, dolphin.ccan, type="split", color="Phylum", shape="DolphinFactor", label="X.SampleID")

p3n

```

# CCA plot with Mammal groups of interest indicated

================================================================

```{r}

Mammal=factor(rep("NotMarine",nsamples(phys)),

levels=c("NotMarine","SeaLion","Seal",

"Dolphin","PolarBear","Manatee","Dugong","Porpoise"))

Mammal[ which(sample_data(phys)[,3]=="AtlanticBottleNoseDolphin")]="Dolphin"

Mammal[ which(sample_data(phys)[,3]=="CaliforniaSealion")]="SeaLion"

Mammal[ which(sample_data(phys)[,3]=="GreySeal")]="Seal"

Mammal[ which(sample_data(phys)[,3]=="Manatee")]="Manatee"

Mammal[ which(sample_data(phys)[,3]=="HarborSeal")]="Seal"

Mammal[which(sample_data(phys)[,3]=="HoodedSealPooled")]="Seal"

Mammal[which(sample_data(phys)[,3]=="PolarBear")]="PolarBear"

Mammal[which(sample_data(phys)[,3]=="YangtzeFinlessPorpoise")]="Porpoise"

Mammal[which(sample_data(phys)[,3]=="Dugong")]="Dugong"

####This is because I am accumulating errors here

sample_data(phys)=sample_data(phys)[,1:26]

sample_data(phys)=data.frame(sample_data(phys),Mammal)

```

```{r}

myshapes=c(16,15,17,3,7,8,10,13,22)

mammal.allcca <- ordinate(phys, "CCA",formula = otu_matrix ~ Diet+Habitat/HostGroup)

anova(mammal.allcca)

pp= plot_ordination(phys,mammal.allcca,type="biplot",shape="Mammal")+

geom_point(size = 5, alpha = 0.5)+scale_shape_manual(values = myshapes)

pp

```

Subsample dolphins/manatees/sea lions to 6 samples each:

============================================

Take out 13 random dolphins and 3 random manatees then redo analysis:

```{r}

dolphins=which(sample_data(phys)[,3]=="AtlanticBottleNoseDolphin")

manatees=which(sample_data(phys)[,3]=="Manatee")

set.seed(0929)

outd=sample(dolphins,13)

outm=sample(manatees,3)

outID=get_variable(phys)[c(outm,outd),1]

lessdolphins=subset_samples(phys,!(get_variable(phys, "X.SampleID") %in% outID))

# Change that one Artiodactyla (Springbok) to Cetartiodactyla

host.order <- sample_data(lessdolphins)$HostOrder

host.order[host.order=="Artiodactyla"] <- "Cetartiodactyla"

host.order <- droplevels(host.order)

sample_data(lessdolphins)$HostOrder <- host.order

```

Make ordinations (Figure 7):

```{r}

ord_NMDS_bray_lessd = ordinate(lessdolphins, "NMDS", "bray")

pBCdiet_ld <- plot_ordination(lessdolphins, ord_NMDS_bray_lessd, color="Diet", title="Mammal Gut (subset dolphins) NMDS Bray Curtis Colored to Diet", label="FigureNumbers") + aes(size=2) + guides(size=FALSE)

pBCdiet_ld$layers[[2]]$geom_params$size <- 3 # Change the label text size

pBCdiet_ld$layers[[2]]$show_guide <- FALSE # Don't put text in the label keys

pBCdiet_ld

pBChabitat_ld <- plot_ordination(lessdolphins, ord_NMDS_bray_lessd, color="Habitat", title="Mammal Gut (subset dolphins) NMDS Bray Curtis Colored to Habitat/Provenance", label="FigureNumbers") + aes(size=2) + guides(size=FALSE)

pBChabitat_ld$layers[[2]]$geom_params$size <- 3 # Change the label text size

pBChabitat_ld$layers[[2]]$show_guide <- FALSE # Don't put text in the label keys

pBChabitat_ld

pBCorder_ld <- plot_ordination(lessdolphins, ord_NMDS_bray_lessd, color="HostOrder", title="Mammal Gut Tip Merged NMDS Bray Curtis Colored to HostOrder", label="FigureNumbers") + aes(size=2) + guides(size=FALSE)

pBCorder_ld$layers[[2]]$geom_params$size <- 3 # Change the label text size

pBCorder_ld$layers[[2]]$show_guide <- FALSE # Don't put text in the label keys

pBCorder_ld

```

Make CCA plot (Figure 8) on the reduced data set:

```{r}

lessd.allcca <- ordinate(lessdolphins, "CCA",formula = otu_matrix ~ Diet+Habitat/HostGroup)

pp= plot_ordination(lessdolphins,lessd.allcca,type="biplot",shape="Mammal") +

geom_point(size = 5, alpha = 0.5)+scale_shape_manual(values = myshapes)

pp

```

Change signs and replot to make it visually more similar to original figure (Figure 8):

```{r}

lessd.allcca$CCA$wa[,1]=(-lessd.allcca$CCA$wa[,1])

lessd.allcca$CCA$wa[,2]=(-lessd.allcca$CCA$wa[,2])

lessd.allcca$CCA$v[,1]=(-lessd.allcca$CCA$v[,1])

lessd.allcca$CCA$v[,2]=(-lessd.allcca$CCA$v[,2])

lessd.allcca$CCA$centroids[,2]=(-lessd.allcca$CCA$centroids[,2])

lessd.allcca$CCA$centroids[,1]=(-lessd.allcca$CCA$centroids[,1])

pp= plot_ordination(lessdolphins,lessd.allcca,type="biplot",shape="Mammal")+

geom_point(size = 5, alpha = 0.5)+scale_shape_manual(values = myshapes)

pp

```

# This code will add position of centroids, change shapes to colors; requires ggvegan

============================================

```{r}

require(vegan)

require(ggplot2)

require(ggvegan)

fmall=fortify(lessd.allcca)

lessd.Mammal <- sample_data(lessdolphins)$Mammal

extracol=factor(rep("NA",nrow(fmall)),levels=c("NA",levels(lessd.Mammal)))

extracol[which(fmall[,3]=="sites")]=lessd.Mammal

fmall=data.frame(fmall,Mammal=extracol)

set.seed(2015)

gp=ggplot(data=fmall,aes(x=Dim1,y=Dim2,label=Label))+

geom_point(data=fmall[fmall[,3]=="sites",],aes(colour = Mammal),size=4,alpha=0.8)+

geom_point(data=fmall[fmall[,3]=="centroids",],shape=17,size=3,vjust=-0.5) +

geom_text(data=fmall[fmall[,3]=="centroids",])+

xlab("CCA1 : 4.5%")+ylab("CC2: 4.1%")

gp=gp+geom_point(data=fmall[fmall[,3]=="species",],shape=18,alpha=0.7,colour="black")

gp

# adding the taxa IDs to the graph - will look ugly but just to help build the Adobe Illustrator plot

# several groups of taxa show heavy overlapping

tp=gp+geom_text(data=fmall[which(fmall[,3]=="species"),],size=3,angle=45,hjust=0.4)

tp

# adding the sample IDs to the graph - will also look ugly, but will be helpful for the Illustrator plot

sp=gp+geom_text(data=fmall[which(fmall[,3]=="sites"),],size=3,angle=45,hjust=0.4)

sp

```
