# Supplementary material for: Marine mammals harbor unique microbiotas shaped by and yet distinct from the sea
Source: Nat Commun. 2016 Feb 3;7:10516. doi: 10.1038/ncomms10516 (PMC4742810; doi:10.1038/ncomms10516)
Supplement: Supplementary Software 4 — R code for analysis of PS sequences obtained from dolphins, sea lions, fish food, and seawater. [file ncomms10516-s5.docx]

**Bik et al., Supplementary Software 4**

**R code for analysis of PS sequences obtained from dolphins, sea lions, fish food, and seawater.**

Authors: Elisabeth Bik, Ben Callahan, Stanford University

**================================================================**

# Background: sample set on 48 dolphins, and 18 sealions. Of 48 dolphins, 10 are wild (from Florida; Location=03FloridaWild), rest managed by MMP in San Diego (most have Location=01SD and some Location=02SatelliteSite)

# Set also include extraction controls (XC), but these have all low numbers of reads and will be removed during processing when we filter for samples >200 reads (see below).

# Specimen types are listed in field SampleTypeSimple and include 01DolOral, 02DolGast, 03DolRect, 05DolChuff, 07DolBlwh, 11SlnOral, 12SlnGast, 13SlnRect, 20Fish, 31Seawater01SD, 32SeawaterSatellite, 35SeawaterFlor.

# Most animals were sampled once. These specimens have SingleTP=1 so if you use that as a selection, you will get 1 timepoint for each animal.

# Some dolphins were sampled monthly for 5 or 6 months, with another timepoint 18 m later. These specimens have TimePoints=1. Note that the first timepoint of those animals is also labeled SingleTP=1

**================================================================**

#### Libraries and initialization:

```{r}

# loading the libraries we need

R.Version()

library("phyloseq")

packageVersion("phyloseq")

library("ggplot2")

packageVersion("ggplot2")

library("ape")

packageVersion("ape")

library("DESeq2")

packageVersion("DESeq2")

library(plotrix)

packageVersion("plotrix")

# source("http://bioconductor.org/biocLite.R")

# biocLite("vsn")

library("plyr")

packageVersion("plyr")

library("doParallel")

packageVersion("doParallel")

library("foreach")

packageVersion("foreach")

theme_set(theme_bw())

# Choose your workspace (change to your own environment)

setwd("/Users/EliesBik/Desktop/")

```

#### Creating a new phyloseq (can skip and import once done)

```{r}

# Import OTU table (healthy animals only)

OTUtable <- import_biom("Merged_EB123_OTU_Table_Selection_Oct2014.biom")

colnames(tax_table(OTUtable)) <- c("Kingdom", "Phylum", "Class", "Order", "Family", "Genus", "Species")

# mapping file for Qiime

mapfile <- import_qiime(mapfilename="EB123_Mapping_Selection_15Jan2015.txt")

# importing a tree

treefile <- import_qiime(treefilename="Merged_EB1_EB2_EB3_rep_set_Selection_Sept2014.tre")

# create a single phyloseq object with a short name

PS <- merge_phyloseq(OTUtable, mapfile, treefile)

print(PS)

# saving the phyloseq object, so next time we can quickly import it

save(PS, file = "DolSL_Selection_Jan2015.RData")

```

# START HERE: Import the data (if already created previously), some light pruning

```{r}

load("DolSL_Selection_Jan2015.RData")

print(PS)

# prune to remove samples under 200 reads

# this will take out the extraction controls as well

ps <- prune_samples(sample_sums(PS)>=200, PS)

print(ps)

# prune to remove low occurrence taxa

# Look at distribution of the number of samples in which each taxa is observed:

otab <- as(otu_table(ps), "matrix") # Taxa are rows

present_absent <- (otab > 0)

nsamples <- apply(present_absent, 1, sum)

hist(log(nsamples), 60)

# now prune the low occurrence taxa

ps <- prune_taxa(nsamples > 5, ps) # Found in at least 5 samples

print(ps)

ps <- prune_taxa(taxa_sums(ps) > 20, ps) # More than 20 reads across all samples

print(ps)

head(taxa_sums(ps), 20)

```

#### Subsets, alpha diversity, beta diversity

```{r}

# make subsets Single Timepoints and plot tree

st <- subset_samples(ps,SingleTP=="1")

print(st)

save(st, file = "DolSL_SingleTP_Jan2015.RData")

plot_tree(st, color = "SampleTypeSimple", justify = "left", size = "Abundance")

# Import already created file, single timepoints

load("DolSL_SingleTP_Jan2015.RData")

print(st)

# Plot basic alpha diversity measures (Sobs, Chao1, Shannon), facetted per method, grouped/colored per SampleTypeSimple (Supplementary Figure 5a).

alphast <- plot_richness(st, x="SampleTypeSimple", color="SampleTypeSimple", measures=c("Observed", "Chao1", "Shannon"), title="PyroSingleTPs") + geom_point(size = 2, alpha = 0.75)

alphast

alphaall <- plot_richness(ps, x="SampleTypeSimple", color="SampleTypeSimple", measures=c("Observed", "Chao1", "Shannon"), title="PyroAllTPs")

alphaall

# beta diversity analysis

# ordination on single timepoint set: Bray Curtis / NMDS (Figure 4)

ord_NMDS_bray = ordinate(st, "NMDS", "bray")

p <- plot_ordination(st, ord_NMDS_bray, shape="Location", color="SampleTypeSimple", title="NMDS Bray Curtis Single TPs over 200")

p

p = p + aes(size=2) + guides(size=FALSE)

p

# facetted ordination

plot_ordination(st, ord_NMDS_bray, color="SampleTypeSimple") + facet_wrap(~SampleGroup)

plot_ordination(st, ord_NMDS_bray, color="AnatomicalSite") + facet_wrap(~AnimalSpecies)

# Creating and exporting the distance matrix (Bray Curtis)

distbray = phyloseq::distance(st, method="bray", type="samples")

distb = as.matrix(distbray)

distb[upper.tri(distb)] = NA

distb

write.table(distb, "DistanceMatrix_DolSL_Bray_July2015.txt", sep="\t", col.names=NA, quote=F)

# make a subset containing only dolphin samples, single timepoints

dol <- subset_samples(st, AnimalSpecies=="Dol")

print(dol)

# ordination of dolphin samples on sample type, age, gender

# Supplementary Figure 8

ord_NMDS_bray_dol = ordinate(dol, "NMDS", "bray")

pSampleType <- plot_ordination(dol, ord_NMDS_bray_dol, color="SampleTypeSimple", title="NMDS Bray Curtis Dolphin Single TPs over 200") + aes(size=2) + guides(size=FALSE)

pSampleType

pGender <- plot_ordination(dol, ord_NMDS_bray_dol, color="Gender", title="NMDS Bray Curtis Dolphin Single TPs over 200") + aes(size=2) + guides(size=FALSE)

pGender

pAge <- plot_ordination(dol, ord_NMDS_bray_dol, color="AgeGroup", title="NMDS Bray Curtis Dolphin Single TPs over 200") + aes(size=2) + guides(size=FALSE)

pAge

pLocation <- plot_ordination(dol, ord_NMDS_bray_dol, color="Location", title="NMDS Bray Curtis Dolphin Single TPs over 200") + aes(size=2) + guides(size=FALSE)

pLocation

pRunID <- plot_ordination(dol, ord_NMDS_bray_dol, color="RunID", title="NMDS Bray Curtis Dolphin Single TPs over 200") + aes(size=2) + guides(size=FALSE)

pRunID

# make dolphin and sealion oral and rectal subsets, ordinate wild vs managed (Figure 5)

maror <- subset_samples(st, AnatomicalSite=="Oral")

print(maror)

ord_NMDS_bray_maror = ordinate(maror, "NMDS", "bray")

pMarOrLoc <- plot_ordination(maror, ord_NMDS_bray_maror, shape="Location", color="SampleTypeSimple", title="NMDS Bray Curtis Dol SL ORAL Single TPs over 200") + aes(size=2) + guides(size=FALSE)

pMarOrLoc

marrc <- subset_samples(st, AnatomicalSite=="Rect")

print(marrc)

ord_NMDS_bray_marrc = ordinate(marrc, "NMDS", "bray")

pMarRcLoc <- plot_ordination(marrc, ord_NMDS_bray_marrc, shape="Location", color="SampleTypeSimple", title="NMDS Bray Curtis Dol SL RECTAL Single TPs over 200") + aes(size=2) + guides(size=FALSE)

pMarRcLoc

```

#### BJC: Bootstrap Test of Dolphin Samples by Location

# This part was written by Ben Callahan to test if oral and rectal specimens are significantly different between MMP and wild dolphins

```{r}

set.seed(100)

deltaBetweenWithin <- function(dmat, cls) {

wtin <- mean(dmat[outer(cls==1, cls==1) | outer(cls==2, cls==2)], na.rm=TRUE)

btwn <- mean(dmat[outer(cls==1, cls==2) | outer(cls==2, cls==1)], na.rm=TRUE)

}

# Dolphin oral samples

#print(maror)

pso <- subset_samples(maror, AnimalSpecies=="Dol")

#print(pso)

pso <- subset_samples(pso, sample_data(pso)$Location != "02SatelliteSite")

#print(pso)

table(sample_data(pso)$Location)

loco <- as.integer(sample_data(pso)$Location)

table(loco)

bco <- as.matrix(phyloseq::distance(pso, method="bray", type="samples"))

bco[upper.tri(bco, diag=TRUE)] <- NA

deltao <- deltaBetweenWithin(bco, loco)

NSAM <- 10000

deltao.boot <- rep(NA, NSAM)

for(i in seq(NSAM)) {

deltao.boot[[i]] <- deltaBetweenWithin(bco, sample(loco))

}

summary(deltao.boot)

deltao

mean(deltao.boot > deltao)

# Dolphin rectal samples

#print(marrc)

psr <- subset_samples(marrc, AnimalSpecies=="Dol")

#print(psr)

psr <- subset_samples(psr, sample_data(psr)$Location != "02SatelliteSite")

#print(psr)

table(sample_data(psr)$Location)

locr <- as.integer(sample_data(psr)$Location)

table(locr)

bcr <- as.matrix(phyloseq::distance(psr, method="bray", type="samples"))

bcr[upper.tri(bcr, diag=TRUE)] <- NA

deltar <- deltaBetweenWithin(bcr, locr)

NSAM <- 10000

deltar.boot <- rep(NA, NSAM)

for(i in seq(NSAM)) {

deltar.boot[[i]] <- deltaBetweenWithin(bcr, sample(locr))

}

summary(deltar.boot)

deltar

mean(deltar.boot > deltar)

```

So, p(oral) < 0.001, and p(rect) = 0.11.

#### BJC: Time stability

# The following part was also written by Ben Callahan. Here, we are looking at the 7 dolphins that were sampled monthly for 5 or 6 months, and an additional time 3 years later. One dolphin (U) was also sampled about 1 year before the monthly sampling.

```{r}

# Prune the phyloseq object down to just the relevant samples from the 7 repeatedly sequenced dolphins

pst <- prune_samples(sample_data(ps)$AnimalSpecies == "Dol", ps)

tab <- table(sample_data(pst)$AnimalID)

keep <- names(tab)[tab>10]

pst <- prune_samples(sample_data(pst)$AnimalID %in% keep, pst)

table(sample_data(pst)$AnimalID, sample_data(pst)$AnatomicalSite)

pst <- prune_samples(sample_data(pst)$AnatomicalSite %in% c("Gast", "Oral", "Rect"), pst)

colnames(sample_data(pst))

any(sample_data(pst)$Replicate)

head(sample_data(pst)$TemporalPoint)

levels(sample_data(pst)$TemporalPoint)

time <- sapply(strsplit(as.character(sample_data(pst)$TemporalPoint), "_"), `[`, 2)

append <- substr(time, nchar(time))

append[append == "0"] <- "M"

append

time <- substr(time, 1, nchar(time)-1)

time[time==""] <- "0"

time <- as.numeric(time)

time[append == "Y"] <- time[append == "Y"]*12

time[sample_data(pst)$AnimalID=="U"] <- time[sample_data(pst)$AnimalID=="U"]-12

time[time==24] <- 36 ### Corrected

time

sample_data(pst)$Time <- time

samdf <- sample_data(pst)

any(duplicated(samdf[,c("AnimalID", "Time", "AnatomicalSite")]))

sum(duplicated(samdf[,c("AnimalID", "Time", "AnatomicalSite")]))

sum(is.na(samdf$TimePoints))

fix <- rownames(samdf)[!is.na(samdf$SingleTP) & is.na(samdf$TimePoints)] # FIX THE TIMEPOINTS COL FOR THESE TWO SAMPLES

sample_data(pst)$TimePoints[sample_data(pst)$X.SampleID %in% fix)] <- 1

pst <- prune_samples(!is.na(sample_data(pst)$TimePoints), pst)

samdf <- data.frame(sample_data(pst))

any(duplicated(samdf[,c("AnimalID", "Time", "AnatomicalSite")]))

pst <- prune_samples(sample_data(pst)$Time >= 0, pst)

sample_data(pst)$Late <- sample_data(pst)$Time > 10

# pst now contains the relevant samples

```

```{r}

# Display the sampling regimen

samdf <- data.frame(sample_data(pst))

psam <- ggplot(data=samdf, aes(x=Time, y=as.numeric(AnimalID)+0.1*as.numeric(AnatomicalSite), color=AnatomicalSite))

psam + geom_point()

```

```{r}

# Make site-specific phyloseq objects

pstG <- prune_samples(sample_data(pst)$AnatomicalSite == "Gast", pst)

pstO <- prune_samples(sample_data(pst)$AnatomicalSite == "Oral", pst)

pstR <- prune_samples(sample_data(pst)$AnatomicalSite == "Rect", pst)

pstGe <- prune_samples(sample_data(pstG)$Late == FALSE, pstG)

pstOe <- prune_samples(sample_data(pstO)$Late == FALSE, pstO)

pstRe <- prune_samples(sample_data(pstR)$Late == FALSE, pstR)

```

```{r}

# Mantel tests for association between same/different dolphin and community dissimilarity for each bodysite

# Only considering the monthly sampling period

bcGe <- as.matrix(phyloseq::distance(pstGe, method="bray"))

samdf <- data.frame(sample_data(pstGe))

sameDol <- Reduce("|", lapply(unique(samdf$AnimalID), function(x) outer(samdf$AnimalID==x, samdf$AnimalID==x)))

mantel(bcGe, 1 * !sameDol, permutations=9999)

#delt <- outer(samdf$Time, samdf$Time, FUN=function(x,y) abs(x-y))

#mantel(bcGe, delt)

bcOe <- as.matrix(phyloseq::distance(pstOe, method="bray"))

samdf <- data.frame(sample_data(pstOe))

sameDol <- Reduce("|", lapply(unique(samdf$AnimalID), function(x) outer(samdf$AnimalID==x, samdf$AnimalID==x)))

mantel(bcOe, 1 * !sameDol, permutations=9999)

#delt <- outer(samdf$Time, samdf$Time, FUN=function(x,y) abs(x-y))

#mantel(bcOe, delt)

bcRe <- as.matrix(phyloseq::distance(pstRe, method="bray"))

samdf <- data.frame(sample_data(pstRe))

sameDol <- Reduce("|", lapply(unique(samdf$AnimalID), function(x) outer(samdf$AnimalID==x, samdf$AnimalID==x)))

mantel(bcRe, 1 * !sameDol, permutations=9999)

#delt <- outer(samdf$Time, samdf$Time, FUN=function(x,y) abs(x-y))

#mantel(bcRe, delt)

```

```{r}

# Show ordination with the +3Y data point (Figure 6)

shpscl <- scale_shape_manual(values=c("TRUE" = 1, "FALSE" = 16))

require(gridExtra)

grid.arrange(plot_ordination(pstG, ordinate(pstG, "NMDS", "bray"), color="AnimalID", shape="Late") + ggtitle("Gast") + shpscl, plot_ordination(pstO, ordinate(pstO, "NMDS", "bray"), color="AnimalID", shape="Late") + ggtitle("Oral") + shpscl, plot_ordination(pstR, ordinate(pstR, "NMDS", "bray"), color="AnimalID", shape="Late") + ggtitle("Rect") + shpscl, nrow=1)

```

```{r}

# Compare the distances of early to late samples w/in and between dolphins

# Have to subset to those dolphin/body-sites that have a late sample

has.late <- tapply(sample_data(pstG)$Late, sample_data(pstG)$AnimalID, any)

has.late <- names(has.late)[has.late]

pstGhl <- prune_samples(sample_data(pstG)$AnimalID %in% has.late, pstG)

has.late <- tapply(sample_data(pstO)$Late, sample_data(pstO)$AnimalID, any)

has.late <- names(has.late)[has.late]

pstOhl <- prune_samples(sample_data(pstO)$AnimalID %in% has.late, pstO)

has.late <- tapply(sample_data(pstR)$Late, sample_data(pstR)$AnimalID, any)

has.late <- names(has.late)[has.late]

pstRhl <- prune_samples(sample_data(pstR)$AnimalID %in% has.late, pstR)

```

```{r}

# Now do comparisons

bc <- as.matrix(phyloseq::distance(pstGhl, method="bray"))

bc[upper.tri(bc, diag=TRUE)] <- NA

samdf <- data.frame(sample_data(pstGhl))

sameDol <- Reduce("|", lapply(unique(samdf$AnimalID), function(x) outer(samdf$AnimalID==x, samdf$AnimalID==x)))

bothEarly <- outer(!samdf$Late, !samdf$Late)

eitherOr <- outer(!samdf$Late, samdf$Late) | outer(samdf$Late, !samdf$Late)

cat("Gast: BothEarly-DiffDol:", mean(bc[!sameDol & bothEarly], na.rm=TRUE), "... BothEarly-SameDol:", mean(bc[sameDol & bothEarly], na.rm=TRUE))

cat("Gast: EarlyLate-DiffDol:", mean(bc[!sameDol & eitherOr], na.rm=TRUE), "... EarlyLate-SameDol:", mean(bc[sameDol & eitherOr], na.rm=TRUE))

bc <- as.matrix(phyloseq::distance(pstOhl, method="bray"))

bc[upper.tri(bc, diag=TRUE)] <- NA

samdf <- data.frame(sample_data(pstOhl))

sameDol <- Reduce("|", lapply(unique(samdf$AnimalID), function(x) outer(samdf$AnimalID==x, samdf$AnimalID==x)))

bothEarly <- outer(!samdf$Late, !samdf$Late)

eitherOr <- outer(!samdf$Late, samdf$Late) | outer(samdf$Late, !samdf$Late)

cat("Oral: BothEarly-DiffDol:", mean(bc[!sameDol & bothEarly], na.rm=TRUE), "... BothEarly-SameDol:", mean(bc[sameDol & bothEarly], na.rm=TRUE))

cat("Oral: EarlyLate-DiffDol:", mean(bc[!sameDol & eitherOr], na.rm=TRUE), "... EarlyLate-SameDol:", mean(bc[sameDol & eitherOr], na.rm=TRUE))

bc <- as.matrix(phyloseq::distance(pstRhl, method="bray"))

bc[upper.tri(bc, diag=TRUE)] <- NA

samdf <- data.frame(sample_data(pstRhl))

sameDol <- Reduce("|", lapply(unique(samdf$AnimalID), function(x) outer(samdf$AnimalID==x, samdf$AnimalID==x)))

bothEarly <- outer(!samdf$Late, !samdf$Late)

eitherOr <- outer(!samdf$Late, samdf$Late) | outer(samdf$Late, !samdf$Late)

cat("Rect: BothEarly-DiffDol:", mean(bc[!sameDol & bothEarly], na.rm=TRUE), "... BothEarly-SameDol:", mean(bc[sameDol & bothEarly], na.rm=TRUE))

cat("Rect: EarlyLate-DiffDol:", mean(bc[!sameDol & eitherOr], na.rm=TRUE), "... EarlyLate-SameDol:", mean(bc[sameDol & eitherOr], na.rm=TRUE))

```
